# A functional loop between YTH domain family protein YTHDF3 mediated m^6^A modification and phosphofructokinase PFKL in glycolysis of hepatocellular carcinoma

**DOI:** 10.1186/s13046-022-02538-4

**Published:** 2022-12-06

**Authors:** Rong Zhou, Wen Ni, Chao Qin, Yunxia Zhou, Yuqing Li, Jianping Huo, Lijuan Bian, Aijun Zhou, Jianming Li

**Affiliations:** 1 Department of Pathology, Sun Yat-sen Memorial Hospital, Sun Yat-sen University, Guangzhou, 510120 China; 2 Guangdong Province Key Laboratory of Malignant Tumor Epigenetics and Gene Regulation, Sun Yat-sen Memorial Hospital, Sun Yat-sen University, Guangzhou, 510120 China; 3 The Seventh Affiliated Hospital of Sun Yat-sen University, Shenzhen, 518107 China

**Keywords:** Hepatocellular carcinoma, YTHDF3, PFKL, mRNA splicing

## Abstract

**Background & aims:**

N^6^-methyladenosine (m^6^A) modification plays a critical role in progression of hepatocellular carcinoma (HCC), and aerobic glycolysis is a hallmark of cancer including HCC. However, the role of YTHDF3, one member of the core readers of the m^6^A pathway, in aerobic glycolysis and progression of HCC is still unclear.

**Methods:**

Expression levels of YTHDF3 in carcinoma and surrounding tissues of HCC patients were evaluated by immunohistochemistry. Loss and gain-of-function experiments in vitro and in vivo were used to assess the effects of YTHDF3 on HCC cell proliferation, migration and invasion. The role of YTHDF3 in hepatocarcinogenesis was observed in a chemically induced HCC model with *Ythdf3*^−/−^ mice. Untargeted metabolomics and glucose metabolism phenotype assays were performed to evaluate relationship between YTHDF3 and glucose metabolism. The effect of YTHDF3 on PFKL was assessed by methylated RNA immunoprecipitation assays (MeRIP). Co-immunoprecipitation and immunofluorescence assays were performed to investigate the connection between YTHDF3 and PFKL.

**Results:**

We found YTHDF3 expression was greatly upregulated in carcinoma tissues and it was correlated with poor prognosis of HCC patients. Gain-of-function and loss-of-function assays demonstrated YTHDF3 promoted proliferation, migration and invasion of HCC cells in vitro, and YTHDF3 knockdown inhibited xenograft tumor growth and lung metastasis of HCC cells in vivo. YTHDF3 knockout significantly suppressed hepatocarcinogenesis in chemically induced mice model. Mechanistically, YTHDF3 promoted aerobic glycolysis by promoting phosphofructokinase PFKL expression at both mRNA and protein levels. MeRIP assays showed YTHDF3 suppressed PFKL mRNA degradation via m^6^A modification. Surprisingly, PFKL positively regulated YTHDF3 protein expression, not as a glycolysis rate-limited enzyme, and PFKL knockdown effectively rescued the effects of YTHDF3 overexpression on proliferation, migration and invasion ability of Sk-Hep-1 and HepG2 cells. Notably, co-immunoprecipitation assays demonstrated PFKL interacted with YTHDF3 via EFTUD2, a core subunit of spliceosome involved in pre-mRNA splicing process, and ubiquitination assays showed PFKL could positively regulate YTHDF3 protein expression via inhibiting ubiquitination of YTHDF3 protein by EFTUD2.

**Conclusions:**

our study uncovers the key role of YTHDF3 in HCC, characterizes a positive functional loop between YTHDF3 and phosphofructokinase PFKL in glucose metabolism of HCC, and suggests the connection between pre-mRNA splicing process and m^6^A modification.

**Supplementary Information:**

The online version contains supplementary material available at 10.1186/s13046-022-02538-4.

## Introduction

Nowadays, liver cancer is the second most common cause of cancer-related death in the world [1]. Hepatocellular carcinoma (HCC) is the most common type of liver cancer. In terms of mortality, HCC is the second in males, and is the third among females in China [2]. As we all know, a large number of factors promote HCC carcinogenesis and progression, including viral hepatitis, excessive alcohol and various metabolic factors like obesity and fatty liver disease. Notably, increasing evidences suggest that alterations of epigenetic modifications including RNA modifications lead to dysfunction of oncogenic and tumor suppressor genes, promoting progression of HCC [3].

N^6^-methyladenosine (m^6^A) modification, the methylation of adenosine at position 6, is one of the best characterized RNA modifications among mRNA and non-coding RNAs in eukaryotic cells. m^6^A modification is a kind of dynamic modification, which can be actively regulated by the core components of m^6^A pathway, including writers, erasers and readers. More and more studies demonstrate that these core components of m^6^A modification play important roles in tumorigenesis and tumor progression among many types of cancer [4]. Among these core components, m^6^A is recognized by readers such as YTH domain family proteins (YTHDF1, YTHDF2, YTHDF3, and YTHDC2), which directly bind m^6^A sites of other complexes to regulate RNA metabolism, like pre-mRNA splicing, RNA folding and protein translation [5]. Interestingly, the roles of YTHDF1 or YTHDF2 in HCC have been explicitly illustrated recently [6, 7], but the role of YTHDF3 in HCC progression is still unclear.

Glycolysis is a crucial metabolic process in cells as it can provide energy quickly when cells are deprived of oxygen. Metabolism reprogramming has been regarded as a hallmark of cancer. In HCC, the transition from oxidative phosphorylation pathway to glycolysis pathway, a rapid but low-efficiency metabolic process, is critical for tumorigenesis and progression of HCC. Notably, cancer cells reprogram metabolic related enzymes to meet their demands, facilitate cancer cells proliferation, and provide a more suitable microenvironment for the survival of cancer cells [8]. However, the relationship between RNA modification and glycolysis, and the role of m^6^A modification among key glycolysis enzymes are still not well-studied.

Here, in our study, we characterized a functional loop between YTHDF3 and phosphofructokinase PFKL in glycolysis metabolism of HCC. We not only identified the role of YTHDF3 in m^6^A modification of PFKL mRNA but also established the unexpected role of phosphofructokinase PFKL in regulation of YTHDF3 and m^6^A modification.

## Materials and methods

### HCC patients’ clinical specimens and data

This study has been approved by the Human Research Ethics Committees of Sun Yat-sen University (SYSKY-2022-120-01). All subjects have been informed of the study details and signed informed consent forms. HCC patients’ samples were collected from Department of Pathology at Sun Yat-sen Memorial Hospital, Sun Yat-sen University (Guangzhou, China) from July 2015 to December 2017 according to the following criteria. Firstly, they were diagnosed with HCC according to the updated diagnostic guidelines. Secondly, they couldn’t have other diseases that affected blood glucose level. Thirdly, they were able to cooperate with our researchers for following up.

Their carcinoma and surrounding tissues were collected and placed in liquid nitrogen, stored at − 80 °C until be used or made into paraffin sections. HCC patients’ clinicopathological characteristics and preoperative biochemical indicators were also collected. After patients discharged from hospital, they were followed up by telephone every 3 months so that we could master the prognosis of them. The end time of follow-up was December 31, 2020.

### Histological analysis and immunohistochemistry (IHC)

The paraffin sections were deparaffinized with xylene and hydrated with gradient alcohol. Then we repaired the antigenic sites of section with EDTA buffer (1 mM, PH8.0) (ZSGB BIO, Beijing, China) by a high-pressure method. The sections were cooled to room temperature, sealed with peroxidase and goat serum successively, and incubated overnight with primary antibody at 4 °C. On the second day, the sections were incubated with secondary antibody at 37 °C for 30 min. We used Diaminobenzidine (DAB) (ZSGB BIO, Beijing, China) to stain the target proteins, and used hematoxylin to stain nucleus. We evaluated IHC staining scores according to the product of staining intensity and percentage of staining area of positive signal. Staining intensity was classified as zero (negative), one (weak), two (moderate) and three (strong) score. Percentage of staining area were classified as zero (< 5%), one (5–25%), two (25–50%), three (50–75%) and four (> 75%) score. The low and high expression group of specific protein was divided according to the median value of total staining score. All antibodies were described in additional files (Supplementary 1).

### Cell culture and transfection

All cell lines, including Sk-Hep-1, SNU449, Huh7, HepG2, MHCC-97H, MHCC-97 L, SMMC-7721 and 293 T cells, were purchased from the Cell Bank of Type Culture Collection (Guangzhou Cellcook Biotech Co., Ltd., Guangzhou, China). According to the requirement of cells, culturing cells with DMEM or RPMI1640 medium containing 10% fetal bovine serum and 1% penicillin-streptomycin in a cell incubator at 37 °C and 5% CO_2_.

According to the background expression of YTHDF3 among HCC cell lines, YTHDF3 overexpression lentivirus were transfected into Sk-Hep-1 and HepG2 cells to construct YTHDF3-stably-overexpressed cell lines. Meanwhile, two short hairpin RNAs (shYTHDF3–1 and shYTHDF3–2) were transfected into Huh7 and SNU449 cells to construct YTHDF3-stably-knocked down cell lines. After 2 weeks, we used puromycin (MP Biomedicals) to screen stably transfected cells. jetPRIME (Polyplus, Strasbourg, France) was used for transiently transfecting cells with plasmids or small interfering RNAs, and we collected cells for other experiments after transfecting for 48 hours.

### RNA isolation and quantitative real-time PCR assay (qPCR)

We extracted total RNA of cells or tissues with Trizol reagent (ThermoFisher). Total RNA was reversed into cDNA with PrimeScript RT Master Mix (TAKARA). qPCR amplifications were performed according to manufacture manual, which is 95 °C for 30s, 95 °C 5 s, 60 °C 20 s for additional 40 cycles. Using β-actin as a control, 2^-ΔΔCt^ method was adopted to calculated the relative mRNA expression of target gene. All primers were described in additional files (Supplementary 1).

### Western blot analysis

Cell precipitate or tissues were fully lysed with middle strength RIPA peptide lysis buffer (Beyotime Biotechnology, Jiangsu, China) containing 1% protein phosphatase inhibitor, 1% protease inhibitor, and 1% phenylmethylsulfonyl fluoride (PMSF), and lysates were centrifuged at 12000 rpm for 10 min at 4 °C. Using BCA protein assay kit (Beyotime Biotechnology, Jiangsu, China) to calculate total protein concentration of supernatant. 20 μg protein of each sample were electrophoresed under a constant voltage (120 V) and were transferred to polyvinylidene fluoride membrane (Millipore) under a constant current (150 mA). Afterwards, the membranes were blocked with 5% fat-free milk for 1 hour, incubated with indicated primary antibodies overnight at 4 °C and incubated with HRP-conjugated secondary antibodies at room temperature for 1 hour successively. We detected the specific protein by using enhanced chemiluminescence reagent. The relative expression of indicated proteins was normalized to β-actin. All antibodies were described in the additional file (Supplementary 1).

### Cell proliferation assay

Cell Counting Kit-8 (CCK-8) assays were used to assess the proliferation capacity of HCC cells. HCC cells at exponential growth stage were cultured into a 96-well plate with 3 × 10^3^ cells/well for 24 hours. CCK-8 reagent (DOJINDO) and completed medium were mixed in a ratio of 1:10. Adding 100 μl mixture to each well. Incubating them at 37 °C for 2 hours under without light conditions and measuring their absorbance values at 450 nm with a microplate reader. We continuously measured them for 5 days and drew the growth curves.

### Colony forming assay

500 HCC cells at exponential growth stage were cultured into each well of 6-well plate with 2 ml complete medium. When visible colonies appeared in the wells, cells were terminated from culture. The colonies were fixed with 4% paraformaldehyde and stained with crystal violet for 30 minutes each. Then washing the plates with running water and drying them naturally. An inverted microscope was used to count visible colonies.

### Wound healing assay

Firstly, 1.5 × 10^6^ cells were cultured into each well of 6-well plate with complete medium containing 10% fetal bovine serum for 24 h to form monolayer cells. Then the monolayer cells were scratched with a 200 μl tip, washed with PBS for three times and cultured in serum-free medium. Afterwards, the wound area was observed and photographed under inverted fluorescence microscope at 0 h, 12 h, 24 h and 48 h after scratching. Image J software was used to calculate wound area of cells at indicated time. The wound healing rate is calculated as (A_0_ - An)/A_0_. A_0_ represents the wound area at 0 h, An represents the wound area at 12 h, 24 h or 48 h. Finally, we draw the wound healing rate curves and compared the HCC cells’ migration capacities.

### Migration and invasion assay

Chambers with Matrigel were used to assess HCC cells’ invasion ability, while chambers without Matrigel were used to assess cells’ migration ability. 200 μl serum-free medium containing 5 × 10^4^ HCC cells were added into upper chambers (8 μm, Corning Inc., USA) and 800 μl medium containing 20% fetal bovine serum was added to the lower chambers. The cells were cultured in an incubator at 37 °C with 5% CO_2_ for 24 hours. Then the upper chambers were fixed with 4% paraformaldehyde and were stained with crystal violet for 30 minutes each. Washing the chambers, capturing images with a forward microscope and counting the HCC cells that invaded or migrated into lower membrane surface at several randomly fields with Image J software.

### In vivo tumorigenicity

All mice experiments were performed according to national and institutional guidelines from the Institutional Animal Care and Use Committees, and have been reviewed and approved by the Animal Ethical and Welfare Committee (AEWC) (Approval No. IACUC-AEWC-F2206018). BALB/c nu/nu male mice (four-week old) were purchased from Laboratory Animal Center, Sun Yat-sen University. All mice were housed in a specific pathogen free environment according to relevant rules.

In the subcutaneous xenograft tumor model, 100 μl mixture of PBS and Matrigel (1:1) (BioCoat) containing 1 × 10^7^ HCC cells were subcutaneously injected into nude mice right flank (5 mice each group). Measuring the maximum and minimum diameters of tumors with a vernier caliper every four days and tumor volume was equal to 0.5xlargest diameterxsmallest diameterxsmallest diameter. After 24 days, the mice were sacrificed and tumors were made into paraffin section or stored at − 80 °C until use.

In the tail vein injection model, 400 μl PBS containing 5 × 10^6^ HCC cells was slowly injected into tail vein of nude mice (5 mice each group). We continued to feed them for four weeks and sacrificed them and collected their lungs, then prepared paraffin sections of lung tissues and stained them with hematoxylin–eosin (HE). The number and total area of metastatic foci were calculated by the software *KFBIO. Slide Viewer*.

We purchased *Ythdf3*^*−/−*^ C57BL/6 mouse which *Ythdf3* genes were knocked out with CRISPR/Cas9 technology. The detail information was elaborated in additional files (Supplementary 2). Heterozygotes were used for mating in order to obtain more male *Ythdf3*^+/+^ and *Ythdf3*^−/−^ mice. One step mouse genotyping kit (Vazyme, PD101) and agarose gel electrophoresis were used to identify the genotype of C57BL/6 mice (Supplementary Fig. 1a). The primers were described in additional files (Supplementary 1). Male *Ythdf3*^+/+^ and *Ythdf3*^−/−^ mice were used to induce HCC model by administration of hepatocarcinogen diethylnitrosamine (DEN, 25 mg/kg, dissolved in PBS) (Rhawn) at 14 days of age, and 0.4 μl/g CCl_4_ (CCl4/olive oil 1:1 (vol: vol)) (Macklin) at 4 weeks old once a week for 28 weeks by intraperitoneal injection. Then they were sacrificed. The mice body weight and liver weight were recorded, the number and maximum diameter of liver tumors were measured, and the liver tissues and plasma were collected for other experiments.

### Metabolites extraction

Samples from *Ythdf3*^*+/+*^ and *Ythdf3*^*−/−*^ mice were thawed on ice. HCC tissues (about 50 mg) were transferred into a 1.5 ml microtube and were cut into small pieces. Extraction steps were as follows: (1) 1 ml methanol: acetonitrile: water (5:3:2) solution was added to precipitate protein and dissolve metabolites with two 5 mm magnetic beads on the high throughout tissue grinder (SCIENTZ-48, China) at 45 Hz for 10 min; (2) transfer the mixture into other microtube, add 500 μl normal saline, vortex for 10 min, and leave aside on ice for 5 min; (3) the mixture was centrifuged at 7800 g for 10 min at 4 °C and 500 μl supernatant was further transferred into other microtube; (4) concentrate the supernatants with a vacuum freeze concentrator.

### UPLC-MS/MS analysis

Each sample was reconstituted by 80 μl methanol and 20 μl H_2_O and centrifuged at 12000 g for 10 min. A 60 μl aliquot of each supernatant was transferred to the auto-sampler vial. 10 μl each rest supernatant was pooled as QC (quality control) group. UPLC-QTOF-MS/MS analysis was performed with a ThermoScientific Ultimate 3000 UPLC couple with Orbitrap Exploris 480 MS in both positive and negative ionization mode using the H-ESI ion source. Samples were injected into a Waters ACQUITY UPLC BEH Amide Column(2.1x100mm, 1.7 μm) with a flow rate of 0.3 ml/min at 40 °C. Before the samples were analyzed, 10 QCs were injected for adjust the system consistency, and one QC was used to estimated system reproducibility and one blank was used to flush the column after each 5 samples were analyzed. The mobile phase consisted of phase A [95:5 (acetonitrile: water) containing 10 mM ammonium formate and 0.1% formic acid] and phase B [50:50 (acetonitrile: water) containing 10 mM ammonium formate and 0.1% formic acid]. The gradient was as follows: solvent B was held constant at 2% for 0.5 min and was increased from 2 to 50% by 12 min, then was deceased to 2% over 14 min, following holding constant in solvent B to 2% at 16 min and then to 98% at 16.1 min, all the method stopped at 23 min. The ion spray voltage was set to 3500 V for positive ion and 3000 V for negative ion mode. Full scan analysis was performed in the electrospray ionization mass spectrometry mode using electrospray ionization technique with coverage of mass range 50-1000 Da by using scan rate of 0.25 s, and the MS/MS screening was accomplished in the combinational mode of positive-information dependent acquisition (IDA) with a scan rate of 0.1 s.

### Glycolysis phenotype assays

To understand how YTHDF3 affects glycolysis in HCC, glucose uptake colorimetric assay kit (Biovision), pyruvate colorimetric assay kit (Biovision), lactate assay kit II (Biovision), ATP colorimetric assay kit (Beyotime Biotechnology, Jiangsu, China) were used. All experiments were performed at least 3 times according to manufacturer’s protocols and all results were normalized to cells number.

For glucose uptake assay, HCC cells at exponential growth stage were seeded into a 96-well plate with 5000 cells/well for 48 hours. Firstly, cells were starved with serum-free medium overnight to increase glucose uptake. Secondly, cells were incubated with 100 μl Krebs-Ringer-Phosphate-HEPES buffer (Ginbio, China) containing 2% BSA for 40 min and were stimulated with 1 μM insulin for 20 min to activate glucose transporter, then 10 μl 2-DG (10 mM) was added to incubate for 20 min. Thirdly, cells were lysed with 80 μl of extraction buffer at 85 °C for 40 min, then we cooled, neutralized, centrifugated the cell lysates at 12000 rpm for 10 min and collected supernatant. After NADPH generation, NADP degradation and recycling amplification reactions, measuring absorbance at 412 nm with microplate reader at 37 °C every 5 min until the 100 pmol standard reached 1.5–2.0 OD.

For pyruvate level assay, 30 μl plasma of each group was directly added into sample wells, while 2 × 10^6^ HCC cells or 10 mg tissues were extracted with pyruvate assay buffer. Then centrifuged (10,000 g, 10 min, 4 °C) to remove insoluble material and collected supernatant. Added 30 μl supernatant and 20 μl pyruvate assay buffer into each well. Then they were incubated for 30 min at room temperature without light, and were measured absorbance at 570 nm with a microplate reader.

For lactate level assay, 20 μl plasma of each group was directly added into sample wells, while 2 × 10^6^ HCC cells or 10 mg tissues were extracted with lactate assay buffer. Then centrifuged (10,000 g, 10 min, 4 °C) to remove all proteins. 20 μl supernatant, 30 μl lactate assay buffer and 50 μl reaction mix were added into each well. Afterwards, incubating the mixture for 30 min at room temperature without light and measured absorbance at 450 nm with a microplate reader.

For ATP level assay, we fully extracted 2 × 10^6^ HCC cells with ATP lysate on ice, centrifuged (12,000 g, 5 min, 4 °C) and collected supernatant. Prepared and added 100 μl ATP detection working solution into per well at room temperature for 3 min so that the background ATP could be consumed. Then add 20 μl supernatant into wells and measured RLU (relative light unit) by using a luminometer.

### Extracellular acidification rate and oxygen consumption rate assays

The extracellular acidification rate (ECAR) and cellular oxygen consumption rate (OCR) were performed with Agilent Seahorse XF Cell Glycolysis Stress Test Kit (Seahorse) and Cell Mito Stress Test Kit (Seahorse) by using the Seahorse XFe96 Extracellular Flux Analyzer (Seahorse Bioscience). Firstly, 1.5 × 10^4^ HCC cells were cultured in seahorse XF cell culture microplates and hydrated a sensor cartridge with ddH_2_O at 37 °C in a non-CO_2_ incubator overnight. Secondly, for the ECAR analysis, prepared assay medium containing 2 mM glutamine, and prepared glucose (10 mmol/L), oligomycin (1.0 μmol/L), and 2-DG (50 mmol/L). For the OCR analysis, prepared assay medium with 1 mM pyruvate, 2 mM glutamine, and 10 mM glucose, and prepared oligomycin (1.5 μM), FCCP (p-trifluoromethoxy carbonyl cyanide phenylhydrazone, 1.0 μM), and rotenone/antimycin A (0.5 μM). Thirdly, these drugs were sequentially injected into each well at indicated time after the baseline measurements by using the analyzer. Finally, the data were collected and analyzed using the XFe96 Wave Software (Agilent).

### RIP assay

Magna RIP Kit (Merck millipore) was used for RIP assay. Sk-Hep-1 and HepG2 cells at exponential growth stage were transiently transfected with 10 μg YTHDF3 plasmids in 10 cm plates for 48 hours, and they were lysed by RIP lysis buffer. RIP lysates were centrifuged and removed 10 μl of supernatant as “input”. Meanwhile, magnetic beads conjugated with anti-YTHDF3 antibody or anti-IgG antibody were prepared. Each rest supernatant and beads-antibody complex were added into RIP immunoprecipitation buffer and incubated overnight at 4 °C. Added proteinase K and 10% SDS and incubated at 55 °C for 30 minutes to digest protein. Then precipitated and purified RNA. Finally, a Nanodrop spectrophotometer was used to measure total RNA or “input”. Quantitative real-time PCR was performed to detect the relative expression of certain genes.

### MeRIP-qPCR

EpiQuik™ CUT&RUN m^6^A RNA Enrichment Kit was used to investigated the m^6^A modification on specific mRNA transcripts. Firstly, 50 μg total RNA was isolated with Trizol reagent from Sk-Hep-1 and Huh7 cells. Secondly, fragments of RNA sequence containing the m^6^A target were cleaved, bound to magnetic beads containing m^6^A antibody, and pulled down. Thirdly, the enriched RNA was released, purified, and eluted. According to the information from SRAMP (http://www.cuilab.cn/sramp), we designed specific primers for YTHDF3 m^6^A-binding proteins. Finally, qPCR was conducted to detect the changes to m^6^A modification of certain genes. All primers were described in additional files (Supplementary 1).

### RNA stability assay

Sk-Hep-1 and HepG2 were transfected with YTHDF3 plasmids, while Huh7 and SNU449 were transfected with siYTHDF3–1, siYTHDF3–2 for 48 hours. Thereafter, these cells were treated with Actinomycin D (ThermoFisher) at 5 μg/ml for 0 h, 3 h, 6 h and 9 h. Total RNA from these cells was extracted with Trizol. The relative expression of certain genes at indicated time was analyzed by quantitative real-time PCR. The degradation rate of mRNA is equal to the ratio of the level of mRNA at indicated time and the level of mRNA at 0 hour. We draw the degradation curve of specific genes on the basis of the degradation rates.

### Co-immunoprecipitation (co-IP)

1.0 × 10^6^ HCC cells or 293 T cells were cultured for 24 hours in 10 cm dishes and they were transfected with 10 μg plasmids for 48 hours. Then they were lysed with weak strength RIPA peptide lysis buffer containing 1% protein phosphatase inhibitor, 1% protease inhibitor, and 1% phenylmethylsulfonyl fluoride (PMSF) for 30 min at 4 °C and were centrifuged at 12000 rpm for 10 min. Collecting supernatant and allocating 10% as “input”. Adding corresponding antibodies into supernatant and incubating them overnight at 4 °C with rotation, then adding agarose A/G beads(1:10) (ThermoFisher) and continuously incubating them at 4 °C for 4–6 hours with rotation. Afterwards, centrifuging them at 3000 rpm for 5 min, discarding supernatant, washing beads with buffer (PBS + 0.5%Triton-100) for five times, resuspending beads with loading buffer (diluted with high strength RIPA peptide lysis buffer), boiling them at 100 °C for 10 min, and centrifuging at 3000 rpm for 5 min. At last, collecting supernatant protein and performing Western blot to verify the interactions between proteins.

### Multi-label immunofluorescence staining

HCC patients’ paraffin sections were deparaffinized with xylene, hydrated with gradient alcohol, and antigen site retrieval with sodium citrate (PH 6.0) in microwave oven for 15 min. The sections were sealed with peroxidase and 5% albumin bovine serum successively. Then they were incubated with EFTUD2 primary antibody for 1 hour at room temperature, washed with PBS for three times, and incubated with secondary antibody (PANOVUE) labeled with horseradish peroxidase (HRP) for 10 min at room temperature. Afterwards, adding fluorescent staining to amplify fluorescent signal for 10 min at room temperature and washing sections with PBS for three times. After that, the antigen sites were repaired by microwave oven again and the above steps were repeated to incubated PFKL and YTHDF3 primary antibody successively. Finally, adding DAPI, sealing sections and collecting images with Vectra Polaris Automated Quantitative Pathology Imaging System.

### Cellular immunofluorescences

1.5 × 10^5^ Sk-Hep-1 cells at exponential growth stage were cultured into confocal dishes for 48 hours. Then they were fixed with 4% paraformaldehyde for 15 min and were washed with PBS for three times. Cellular membrane was broken with 0.5% Triton-100 for 10 min. The non-specific antigen sites were blocked with goat serum for 30 min at room temperature. Cells were incubated overnight with primary antibody at 4 °C, and were incubated with secondary antibody labeled with fluorescent tags for 30 min without light at room temperature. Finally, adding DAPI and taking pictures with Zeiss LSM 800 with airy scan system. All antibodies were described in the additional file (Supplementary 1).

### Ubiquitination assays

Ubiquitination assays of SNU449 cells co-transfected ubiquitin, YTHDF3 and PFKL plasmids, or ubiquitin, YTHDF3 and EFTUD2 plasmids, or ubiquitin, YTHDF3, PFKL and EFTUD2 plasmids by using jetPRIME according to the manufacturer’s instructions (Polyplus, Strasbourg, France) for 24 hours. 20 nM MG-132 was added to RPMI1640 culture medium and cells were incubated continuously for 8 hours. Then, the cells were lysed with weak strength RIPA peptide lysis buffer containing 1% protein phosphatase inhibitor, 1% protease inhibitor, and 1% phenylmethylsulfonyl fluoride (PMSF). Supernatant were incubated with indicated primary antibody overnight with rotation, and incubated with agarose A/G beads(1:10) (ThermoFisher) continuously for 4–6 hours at 4 °C. The eluted proteins were detected by western blotting.

### Statistical analysis

Each assay was performed at least three independent experiments. SPSS 21.0 and GraphPad Prism 8.0 softwares were used. Mean ± SD was used for the quantitative data. The *t* test, *t’* test and *Wilcoxon* test were chosen according to normality and variance homogeneity of the two independent groups, while *one-way ANOVA* analysis was used among three independent groups. *Chi-square* test was used for the qualitative data. Overall survival curves were plotted by *Kaplan-Meier* method and were compared by the log-rank test. *Pearson* correlation was used to evaluated the relationship between two continuous variables. *P* < 0.05 was considered statistically significant. Image J software was used to qualified the green, red and yellow fluorescence co-localization signal intensity, and calculated relative Pearson’s and Mander’s coefficients.

## Results

### YTHDF3 is overexpressed in tumor samples from HCC patients and high expression of YTHDF3 is correlated with poor prognosis of HCC patients

**Fig. 1:**
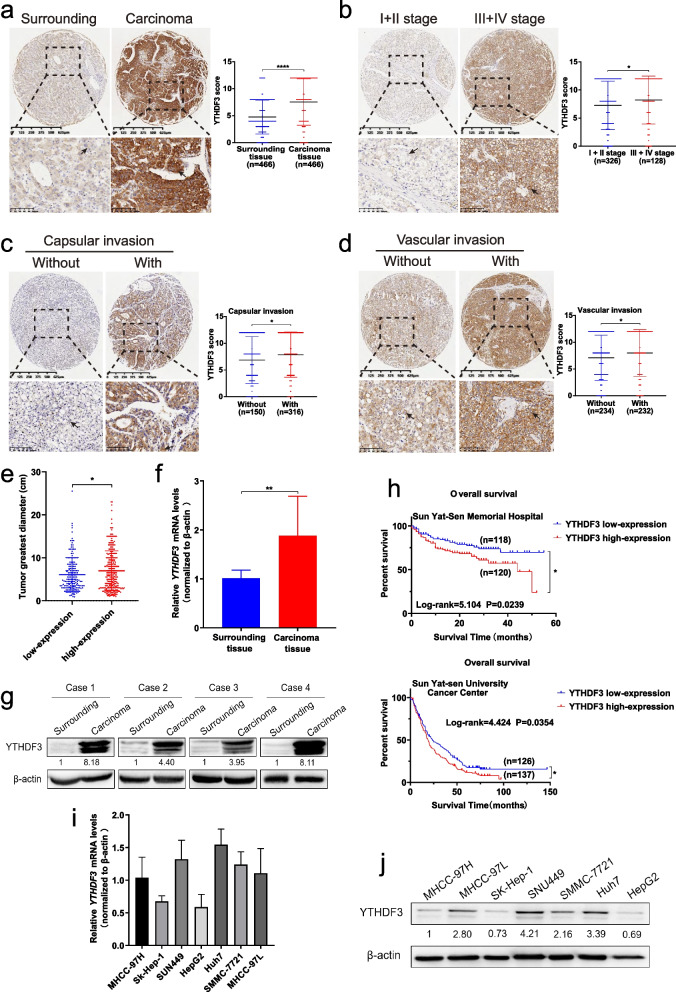
YTHDF3 is overexpressed in tumor samples from HCC patients and high expression of YTHDF3 is correlated with poor prognosis of HCC patients. **a** Representative immunohistochemical images were shown (left) and YTHDF3 protein expression between surrounding and carcinoma tissues of HCC patients (*n* = 466) (right). Scale bar 100 μm. **b** Representative immunohistochemical images were shown (left) and YTHDF3 protein expression between HCC patients in early and middle-late stages (*n* = 464) (right). Scale bar 100 μm. **c** Representative immunohistochemical images were shown (left) and YTHDF3 protein expression between HCC patients without or with capsular invasion (n = 466) (right). Scale bar 100 μm. **d** Representative immunohistochemical images were shown (left) and YTHDF3 protein expression between HCC patients without or with vascular invasion (n = 466) (right). Scale bar 100 μm**. e** Scatter plot analysis of tumor greatest diameters between YTHDF3 low and high-expression HCC patients. **f-g** The relative mRNA and protein expression of YTHDF3 in fresh tumor and surrounding tissues of HCC patients. Normalized to β-actin. **h** Overall survival analysis of HCC patients with low or high expression of YTHDF3 in Sun Yat-Sen Memorial Hospital (upper) and Sun Yat-Sen University Cancer Center (lower). **i** The background mRNA and protein expression of YTHDF3 in HCC cell lines. Normalized to β-actin. Data are shown as mean ± SD (*p < 0.05, **p < 0.01, ***p < 0.001 and ****p < 0.0001). Arrows indicate the location of positive signal of YTHDF3 protein

In order to investigate the role of YTHDF3 in HCC, we performed immunohistochemistry (IHC) of paraffin sections from carcinoma and surrounding tissues of 466 patients diagnosed with HCC from July 2015 to December 2017 to study protein expression of YTHDF3 in HCC. We found protein expression of YTHDF3 in carcinoma tissues was greatly higher than that in normal counterpart surrounding tissues (Fig. 1a). Meanwhile, Western blot and qPCR assays showed that expressions of YTHDF3 in carcinoma tissues were greatly higher than those in normal counterpart surrounding tissues at both mRNA and protein levels (Fig. 1f and g). To further study the relationship between YTHDF3 expression and clinicopathological characteristics of HCC patients, 466 HCC patients were divided into YTHDF3 low-expression (IHC score<8) and high-expression group (IHC score ≥ 8) according to IHC median score of carcinoma tissue. We found that high YTHDF3 expression were correlated with larger tumor diameter (Fig. 1e), capsular invasion (Fig. 1c), vascular invasion (Fig. 1d) or the middle-late stage (Fig. 1b) of HCC patients (Supplementary Table 1).

Importantly, to study whether high YTHDF3 expression level can predict the prognosis of HCC patients, we followed up HCC patients from two cohorts and the end time of follow-up was December 31, 2020. The Kaplan–Meier analysis of 238 HCC patients from Sun Yat-sen Memorial Hospital indicated overexpression of YTHDF3 was correlated to poorer overall survival of HCC patients (log-rank = 5.104, *P* = 0.0239), and the median survival time of the YTHDF3 low-expression group was about 42.85 months, which was apparently longer than that in YTHDF3 high-expression group (about 33.95 months) (Fig. 1h upper). To further identify the prognosis value of YTHDF3 in HCC, the Kaplan–Meier analysis was performed in 263 HCC patients from Sun Yat-Sen University Cancer Center. The median survival time of YTHDF3 low-expression group was about 44.02 months, while the median survival time of YTHDF3 high-expression group was about 28.53 months (Fig. 1 h lower), supporting that overexpression of YTHDF3 predicts poor prognosis of HCC patients.

### YTHDF3 promotes proliferation, migration and invasion of HCC cells in vitro and tumor growth and lung metastasis of HCC in vivo

In order to study the role of YTHDF3 in proliferation, migration, invasion and metastasis ability of HCC cells, we performed a series of gain-of-function and loss-of-function experiments in vitro and in vivo. Firstly, we chose Sk-Hep-1 and HepG2 to establish YTHDF3-stably-overexpressed cell lines, and chose Huh7 and SNU449 to establish YTHDF3-stably-knockdown cell lines according to the background mRNA and protein levels of YTHDF3 in the seven HCC cell lines (Fig. 1i and j).

**Fig. 2:**
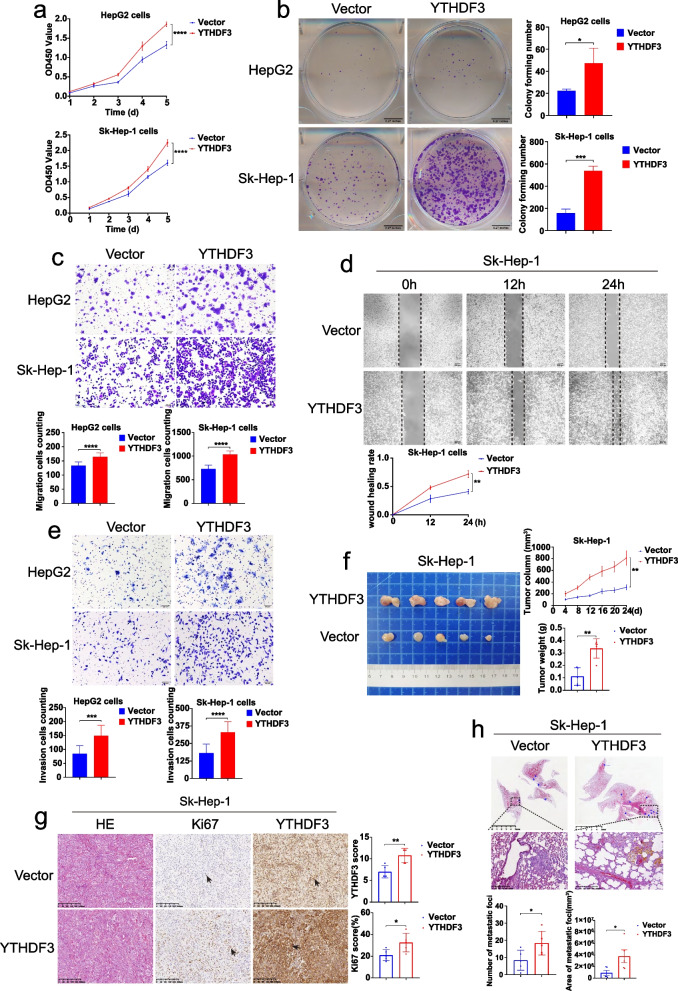
YTHDF3 overexpression promotes proliferation, migration and invasion of Sk-Hep-1 and HepG2 cells in vitro and tumor growth and lung metastasis of Sk-Hep-1 in vivo*.***a-b** CCK8 proliferation assays and colony formation assays were performed to determine cell proliferation of Sk-Hep-1 and HepG2 after YTHDF3 overexpression. **c-d** Transwell migration assays and wound healing assays were performed to determine cell migration of Sk-Hep-1 and HepG2 after YTHDF3 overexpression. The Image J software was used to quantified cells’ migration ability. Scale bar 200 μm. **e** Transwell invasion assays were performed to determine cell invasion of Sk-Hep-1 and HepG2 after YTHDF3 overexpression. The Image J software was used to quantified cells’ invasion ability. Scale bar 200 μm. **f** Representative images of tumor growth in xenografted BALB/c nude mice with Sk-Hep-1 overexpression YTHDF3 cells (left). The growth curves and the average weight of xenograft tumors were shown (right). **g** Representative images of HE and IHC of YTHDF3 and Ki67 of xenograft tumor (left), IHC scores of YTHDF3 and Ki67 were shown in bar graphs (right). Scale bar 200 μm. **h** Representative images of HE staining of orthotopic lung metastasis model (upper). The number and total area of metastatic foci were shown in bar graphs (lower). Scale bar 5 mm. Data are presented as mean ± SD (*p < 0.05, **p < 0.01, ***p < 0.001 and ****p < 0.0001). Arrows indicate the location of positive signal of YTHDF3 or Ki67 protein

CCK8 proliferation assay, colony formation assay, transwell migration assay, wound healing assay and transwell invasion assay showed that overexpression YTHDF3 significantly enhanced proliferation, (Fig. 2a and b), migration (Fig. 2c and d) and invasion (Fig. 2e) ability of Sk-Hep-1 and HepG2 cells in vitro. Moreover, in subcutaneous tumor growth models of mice, YTHDF3 overexpression in Sk-Hep-1 cells facilitated tumor growth in vivo characterized as increasing tumor size and weight (Fig. 2f), and the percentage of Ki67 IHC staining (Fig. 2g), as compared with control group. In addition, orthotopic lung metastasis model of mice demonstrated that the number and total area of metastatic foci in lungs were significantly increased in upregulated YTHDF3 group compared with those in control group (Fig. 2h). In general, YTHDF3 overexpression promoted proliferation, migration and invasion of Sk-Hep-1 and HepG2 cells in vitro and tumor growth and lung metastasis of HCC in vivo*.*

**Fig. 3:**
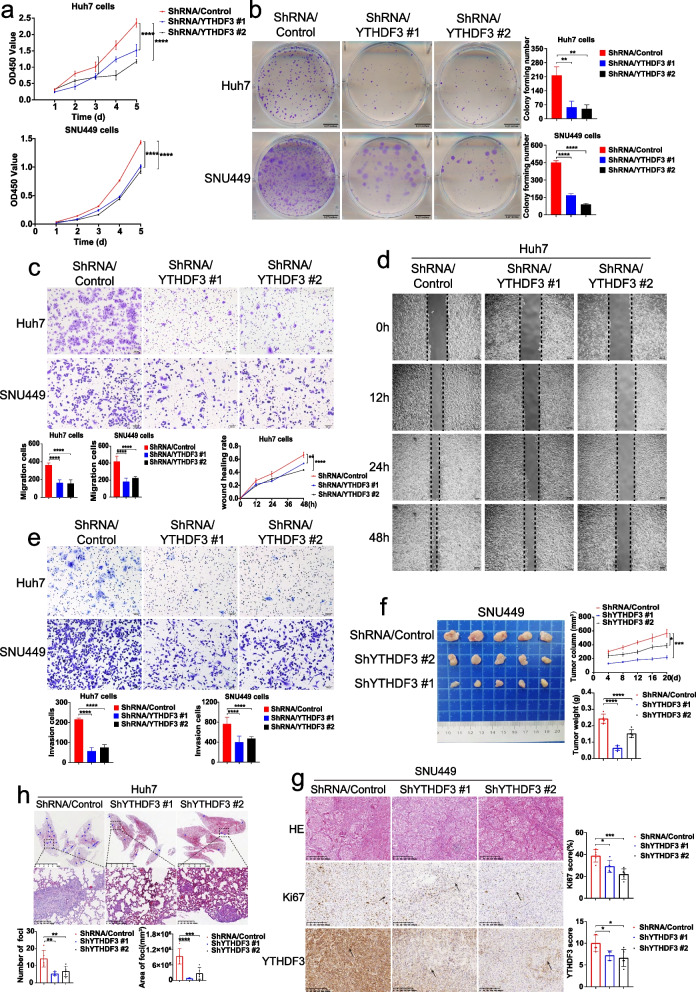
YTHDF3 knockdown inhibits proliferation, migration and invasion of Huh7 and SNU449 cells in vitro and tumor growth and lung metastasis of Huh7 and SNU449 cells in vivo. **a-b** CCK8 proliferation assays and colony formation assays were performed to determine cell proliferation of Huh7 and SNU449 cells after YTHDF3 knockdown. **c-d** Transwell migration assays and wound healing assays were performed to determine cell migration of Huh7 and SNU449 cells after YTHDF3 knockdown. The Image J software was used to quantified cells’ migration ability. Scale bar 200 μm. **e** Transwell invasion assays were performed to determine cell invasion of Huh7 and SNU449 cells after YTHDF3 knockdown. The Image J software was used to quantified cells’ invasion ability.Scale bar 200 μm. **f** Representative images of tumor growth in xenografted BALB/c nude mice with SNU449 knockdown YTHDF3 cells (left). The growth curves and the average weight of xenograft tumors were shown (right).**g** Representative images of HE and IHC staining of YTHDF3 and Ki67 of xenograft tumor (left), IHC scores of YTHDF3 and Ki67 were shown in bar graphs (right). Scale bar 200 μm. **h** Representative images of HE staining of orthotopic lung metastasis model (upper). The number and total area of metastatic foci were shown in bar graphs (lower). Scale bar 5 mm. Data are presented as mean ± SD (*p < 0.05, **p < 0.01, ***p < 0.001 and ****p < 0.0001). Arrows indicate the location of positive signal of YTHDF3 or Ki67 protein

To further determine the functions of YTHDF3 in HCC cells, we established YTHDF3-stably-knockdown cells with Huh7 and SNU449 by using a lentiviral shRNA technique. CCK8 proliferation assay, colony formation assay, transwell migration assay, wound healing assay and transwell invasion assay indicated that YTHDF3 knockdown significantly inhibited proliferation (Fig. 3a and b), migration (Fig. 3c and d), invasion ability (Fig. 3e) of Huh7 and SNU449 cells in vitro. Subcutaneous tumor growth model of mice demonstrated that YTHDF3 knockdown suppressed xenografted tumor growth, tumor weight, and tumor size of HCC cells in vivo (Fig. 3f and g). Meanwhile, in vivo, lung metastasis model of mice demonstrated that the number and total area of metastatic foci in lungs were clearly decreased in YTHDF3-knockdown groups compared with those in control groups (Fig. 3h).

Taken together, gain-of-function and loss-of-function assays uncovered that YTHDF3 promotes proliferation, migration and invasion of HCC cells in vitro and tumor growth and lung metastasis of HCC in vivo*.*

### YTHDF3 knockout suppresses chemically induced hepatocarcinogenesis in mice

**Fig. 4:**
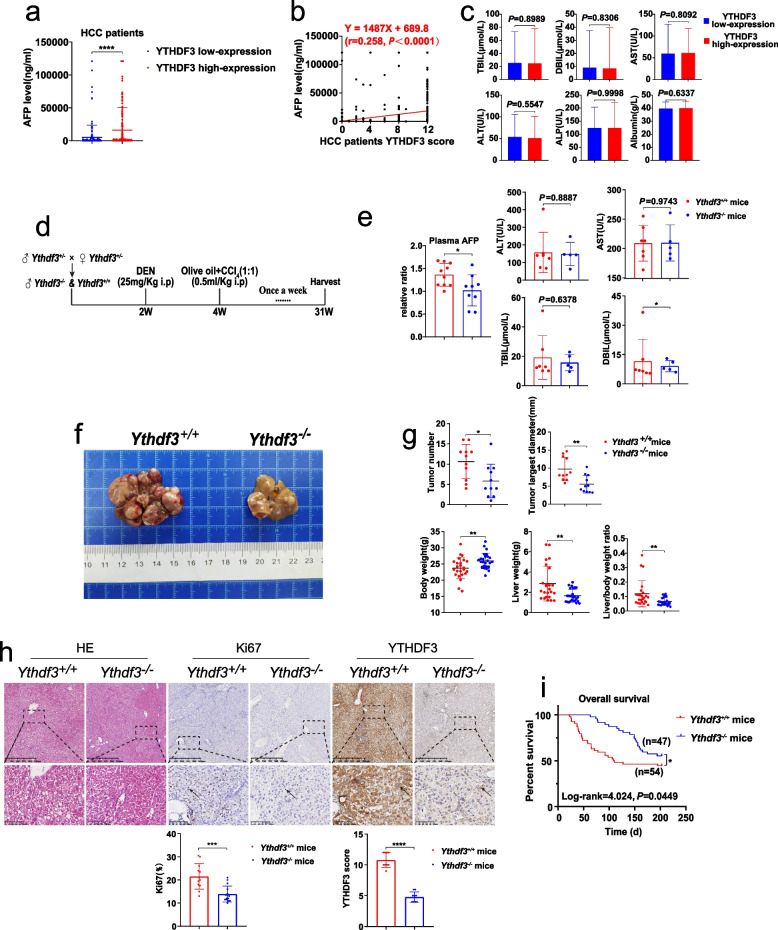
YTHDF3 knockout suppresses chemically induced hepatocarcinogenesis in mice. **a** The serum AFP level comparison between YTHDF3 low and high-expression HCC patients. **b** Scatter plot of correlation analysis between serum AFP level and YTHDF3 IHC score of HCC patients. **c** Histograms of biochemical indicators between YTHDF3 low and high-expression HCC patients. **d** DEN and CCl_4_-induced HCC mouse model flow chart. **e** Comparison of serum APF and biochemical indicators level between *Ythdf3*^+/+^ and *Ythdf3*^−/−^ mice. **f** Gross appearances of representative liver tumors in *Ythdf3*^+/+^ and *Ythdf3*^−/−^ mouse. **g** Comparison of tumor number, tumor largest diameter, body weight, liver weight and liver/body weight ratio between *Ythdf3*^+/+^ and *Ythdf3*^−/−^ mice. **h** Representative images of HE and IHC of YTHDF3 and Ki67 from mice liver tumor tissues of *Ythdf3*^+/+^ and *Ythdf3*^−/−^ mice (upper). IHC scores of YTHDF3 and Ki67 were shown in bar graphs (lower). Scale bar 100 μm. **i** Overall survival analysis of DEN and CCl_4_-induced HCC mice between *Ythdf3*^+/+^ and *Ythdf3*^−/−^ mice. Data are presented as mean ± SD (*p < 0.05, **p < 0.01, ***p < 0.001 and ****p < 0.0001)**.** Abbreviations: IHC immunohistochemistry, HE hematoxylin and eosin staining, AST aspartate transaminase, ALT alanine transaminase, ALP alkaline phosphatase, TBIL total bilirubin, DBIL direct bilirubin. Arrows indicate the location of positive signal of YTHDF3 or Ki67 protein

Interestingly, when we divided HCC patients into YTHDF3 low-expression group and YTHDF3 high-expression group according to IHC staining scores of carcinoma tissue**,** we found that serum AFP level was significantly elevated in YTHDF3 high-expression group (Fig. 4a). Meanwhile, there was a positive correlation between serum AFP level and the expression of YTHDF3 in carcinoma tissue (*r* = 0.258, *P* < 0.0001) (Fig. 4b and c, Supplementary Table 2). These results suggested the potential role of YTHDF3 in carcinogenesis of HCC.

To further study the role of YTHDF3 in hepatocarcinogenesis, we established YTHDF3 knockout (*Ythdf3*^*−/−*^*)* mice by CRISPR/Cas9 technology (Supplementary 2). Heterozygotes were used for mating in order to obtain more male *Ythdf3*^−/−^ mice. Genotypic identification results were performed (Supplementary Fig. 1a). Mouse hepatocarcinogenesis model was induced with *Ythdf3*^+/+^ and *Ythdf3*^−/−^ male mice by the administration of diethylnitrosamine (DEN) at 14 days old and CCl_4_ at 4 weeks old (once a week for 28 weeks) (Fig. 4d). In DEN and CCl_4_ induced HCC mice, we found the number, largest diameter of tumors, liver weight and tumor/body weight ratio of *Ythdf3*^*−/−*^ mice were significantly decreased, compared with those of control *Ythdf3*^+/+^ mice (Fig. 4f and g). Immunohistochemistry staining showed that the expression of Ki67 of liver tumor from *Ythdf3*^*−/−*^ mice was significantly decreased, compared with that from *Ythdf3*^+/+^ control mice (Fig. 4h). Moreover, in DEN and CCl_4_ induced HCC mouse model, Kaplan–Meier analysis indicated that *Ythdf3*^*−/−*^ mice had better overall survival time (log-rank = 4.024, *P* = 0.0449) than that of control wild-type mice. The median survival time of *Ythdf3*^*−/−*^ mice was about 170.85 days, while the median survival time of *Ythdf3*^+/+^ mice was about 126.13 days (Fig. 4i). Accordingly, in DEN and CCl_4_ induced HCC mice, we found that serum AFP level of *Ythdf3*^+/+^ mice was significantly higher than that of *Ythdf3*^*−/−*^ group (Fig. 4e).

In summary, our data indicated that YTHDF3 knockout suppresses chemically induced hepatocarcinogenesis in mice.

### YTHDF3 promotes glycolysis metabolism of HCC cells

**Fig. 5:**
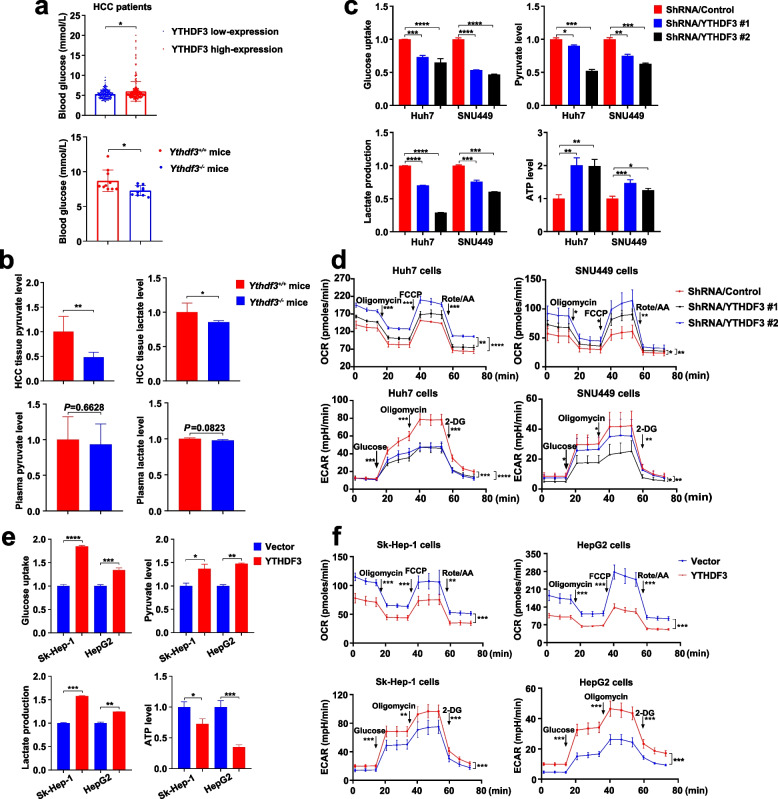
YTHDF3 promotes glycolysis metabolism of HCC cells*.***a** The blood glucose level between YTHDF3 low and high-expression HCC patients (upper). The blood glucose level between *Ythdf3*^*+/+*^ and *Ythdf3*^*−/−*^ mice (lower). **b**. The relative ratio of pyruvate and lactate level in liver cancer tissues and plasma between *Ythdf3*^*+/+*^ and *Ythdf3*^*−/−*^ mice. **c** The relative ratio of glucose uptake, pyruvate, lactate and ATP production after YTHDF3 knockdown in Huh7 and SNU449 cells. **d** ECAR and OCR were examined after YTHDF3 knockdown in Huh7 and SNU449 cells. **e** The relative ratio of glucose uptake, pyruvate, lactate and ATP production after YTHDF3 overexpression in Sk-Hep-1 and HepG2 cells. **f** ECAR and OCR were examined after YTHDF3 overexpression in Sk-Hep-1 and HepG2 cells. Data are presented as mean ± SD (*p < 0.05, **p < 0.01, ***p < 0.001 and ****p < 0.0001)**.** Abbreviations: ECAR, extracellular acidification rate; OCR, cellular oxygen consumption rate

Our study showed that YTHDF3 plays a key role in tumorigenesis and progression of HCC in vitro and in vivo. However, the function of YTHDF3 in glycolysis metabolism remains unclear. We compared the global metabolic profiles of HCC tissues from *Ythdf3*^*+/+*^ and *Ythdf3*^*−/−*^ mice with untargeted metabolomics. In the results, we found that YTHDF3 knockout resulted in disorders of energy metabolic pathways, including glycolysis pathway (Supplementary Fig. 2.). What’s more, when we divided HCC patients into YTHDF3 low-expression group and YTHDF3 high-expression group according to IHC staining scores of carcinoma tissue**,** we found that blood glucose level of HCC patients in high-expression YTHDF3 group was much higher than that in low-expression YTHDF3 group. Moreover, the blood glucose level of *Ythdf3*^*−/−*^ mice was greatly lower than that of *Ythdf3*^*+/+*^ mice (Fig. 5a). To investigate the relationship between YTHDF3 and glucose metabolism in HCC, we detected pyruvate and lactate levels of tumor tissue and plasma from chemically induced hepatocarcinogenesis mice. We found that pyruvate and lactate level in tumor tissue were substantially decreased in *Ythdf3*^*−/−*^ mice (Fig. 5b), compared with those in *Ythdf3*^*+/+*^ mice, suggesting that YTHDF3 promotes growth and progression of HCC by glucose metabolism pathway.

To further establish the relationship between YTHDF3 and glucose metabolism in HCC, we performed a series of experiments to study glucose metabolism including glucose uptake assay, pyruvate and lactate level assay, ATP level assay after YTHDF3 knockdown or overexpression in indicated HCC cells. We found YTHDF3 knockdown in Huh7 and SNU449 cells inhibited glucose uptake, led to decreased levels of pyruvate and lactate and increased level of ATP (Fig. 5c). Moreover, YTHDF3 knockdown decreased extracellular acidification rate (ECAR, reflecting overall glycolytic flux) and increased cellular oxygen consumption rate (OCR, an indicator of mitochondrial oxidative respiration) in Huh7 and SNU449 cells (Fig. 5d). On the other hand, YTHDF3 overexpression promoted glucose uptake, led to increased levels of pyruvate and lactate and decreased level of ATP in Sk-Hep-1 and HepG2 cells (Fig. 5e), as well as the increased ECAR and decreased OCR (Fig. 5f).

In a word, YTHDF3 promotes glycolysis metabolism of HCC cells.

### YTHDF3 promotes phosphofructokinase PFKL expression at both mRNA and protein levels

**Fig. 6:**
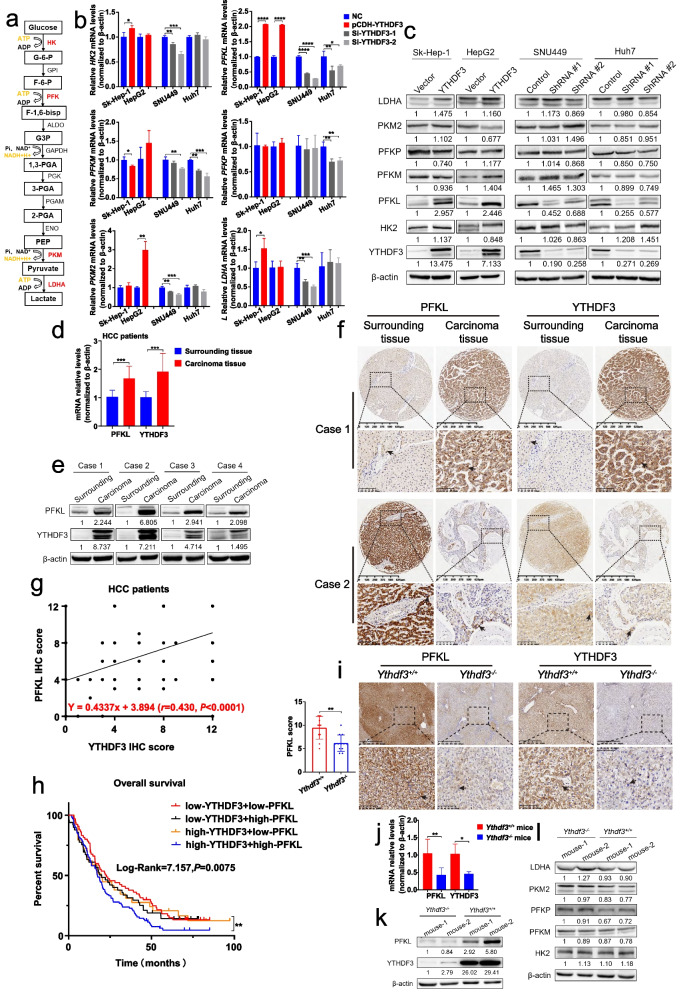
YTHDF3 promotes phosphofructokinase PFKL expression at both mRNA and protein levels. **a** Schematic diagram of glycolysis pathway was shown. **b-c** The relative mRNA and protein expression of rate-limited enzymes of glycolysis after overexpression and knocking down YTHDF3 in indicated HCC cells. **d-e** The relative mRNA and protein expression of PFKL and YTHDF3 from fresh surrounding and carcinoma tissues of HCC patients. **f** Representative immunohistochemistry images of high (or low) expression of PFKL in HCC patients with high (or low) expression of YTHDF3. Scale bar 100 μm. **g** Scatter plot of correlation analysis between PFKL and YTHDF3 IHC score of HCC patients’ carcinoma tissues. **h** Overall survival analysis between four groups HCC patients who were divided by the expression of YTHDF3 and PFKL (including low-expression YTHDF3 + low-expression PFKL, low-expression YTHDF3 + high-expression PFKL, high-expression YTHDF3 + low-expression PFKL, and high-expression YTHDF3 + high-expression PFKL). **i**. Representative immunohistochemistry images of YTHDF3 and PFKL from DEN and CCl_4_-induced HCC mice liver tumor tissues. Scale bar 100 μm. **j-k**. The relative mRNA and protein expression of YTHDF3 and rate-limited enzymes of glycolysis from DEN and CCl_4_-induced HCC mice liver tumor tissues. Data are presented as mean ± SD (*p < 0.05, **p < 0.01, ***p < 0.001 and ****p < 0.0001)**.** Arrows indicate the location of positive signal of YTHDF3 or PFKL protein

Many evidences show high rates of glucose metabolism and glycolysis are critical for cancer cells. However, the mechanisms by which glucose metabolism is reprogrammed in cancer cells are still unclear. To explore how YTHDF3 regulates glycolysis, we studied expression of mRNA and protein of rates-limited enzymes of glycolysis, including hexokinase 2 (HK2, catalyzing glucose to glucose 6-phosphate), phosphofructokinase-1 (PFK1, catalyzing fructose 6-phosphate to fructose 1,6-bisphosphate), pyruvate kinase (PKM, catalyzing phosphoenolpyruvate to pyruvate) and lactic dehydrogenase (LDH, catalyzing pyruvate to lactate) after YTHDF3 knockdown or overexpression in HCC cells. Notably, there are three isoforms of PFK1 in mammals, including PFK-M (found in muscle), PFK-P (found in plasma) and PFK-L (found in liver), and the proportion of these isoforms varies in different tissues depending on their different metabolism requirements (Fig. 6a). Surprisingly, we found that YTHDF3 overexpression resulted in increasing PFKL expressions at mRNA and protein levels in Sk-Hep-1 and HepG2 cells. Moreover, we found that YTHDF3 knockdown led to decreasing PFKL expressions at both mRNA and protein levels in Huh7 and SNU449 cells (Fig. 6b and c).

**Fig. 7:**
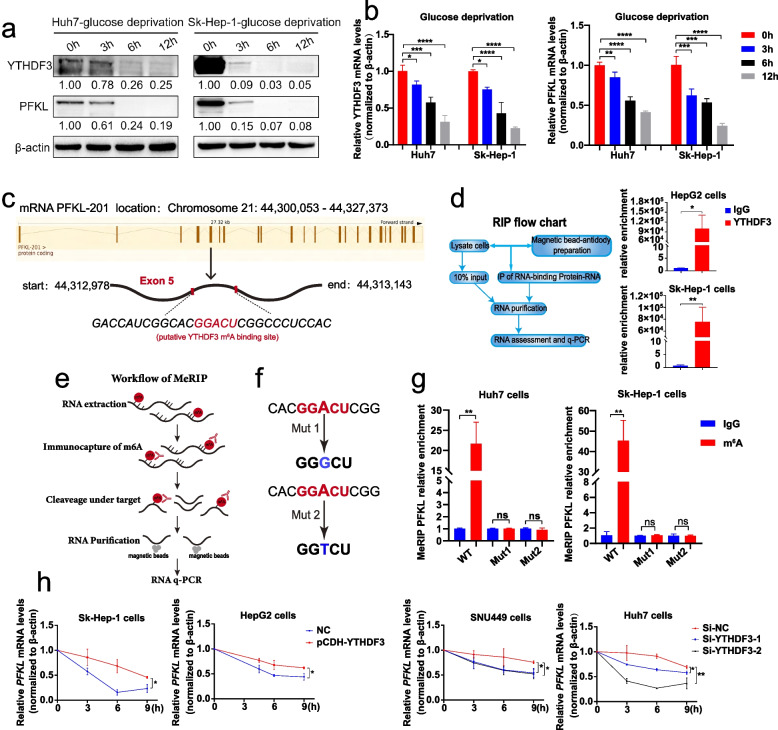
YTHDF3 suppressed the degradation of PFKL mRNA via m^6^A modification. **a-b** The relative mRNA and protein expression of YTHDF3 and PFKL under glucose deprivation condition for 0 h, 3 h, 6 h and 12 h in Huh7 and Sk-Hep-1 cells. (We cultured Huh7 and Sk-Hep-1 cells with glucose-free RPMI1640 medium for 0 h, 3 h, 6 h and 12 h)**. c** Putative YTHDF3 m^6^A modified site to PFKL mRNA transcript according to information of SRAMP website. **d** The RIP- qPCR assay flow chat (left) and the relative enrichment folds of PFKL mRNA correlated with YTHDF3 in Sk-Hep-1 and HepG2 YTHDF3-stably-overexpressed cell lines (right). **e** Workflow of MeRIP- qPCR assay. **f** Two mutant PFKL plasmids used for MeRIP assays. Mut1: adenosine bases (A) in predicted m^6^A binding sites were replaced by guanine base (G). Mut2: adenosine bases (A) in predicted m^6^A binding sites were replaced by thymine base (T). **g** The relative enrichment folds of m^6^A modified PFKL mRNA by YTHDF3 with wild-type PFKL plasmid and two mutant PFKL plasmids in Sk-Hep-1 and Huh7 cells. **h**. PFKL mRNA stability analysis in HCC cells with YTHDF3 transiently knockdown or overexpression in the presence of actinomycin D (5 μg/ml) for 0 h, 3 h, 6 h and 9 h

Interestingly, in clinical patients of HCC, q-PCR and Western blot results of fresh surrounding and carcinoma tissues indicated that high expressions of PFKL were largely associated with high expressions of YTHDF3 at both mRNA and protein levels (Fig. 6d and e). In addition, IHC staining among 456 paraffin sections of HCC patients showed that high expression of PFKL was associated with high expression of YTHDF3 while low expression of PFKL was associated with low expression of YTHDF3 (Fig. 6f). Moreover, there was a positive correlation between PFKL IHC score and YTHDF3 IHC score (*r* = 0.430, *P* < 0.0001) (Fig. 6g). Regarding of overall survival analysis, the Kaplan–Meier analysis indicated that HCC patients with both low expressions of YTHDF3 and PFKL had better survival time than those patients with both high expressions of YTHDF3 and PFKL (log-rank = 7.157, *P* = 0.0075) (Fig. 6h). What’s more, we performed q-PCR, Western blot and IHC with DEN and CCl_4_-induced HCC mice liver tumor tissues. These assays demonstrated that mRNA and protein expression levels of PFKL in liver tumor tissues among *Ythdf3*^*−/−*^ mice were significantly lower than those in *Ythdf3*^+/+^ mice (Fig. 6i, j, k, l). Finally, we cultured Huh7 and Sk-Hep-1 cells with glucose-free RPMI1640 medium for 0 h, 3 h, 6 h and 12 h, and performed Western Blot and qPCR assays with these cells. We found that the mRNA and protein expression of YTHDF3 and PFKL were decreased significantly under glucose deprivation condition (Fig. 7a and b).

Besides these observation, IHC staining showed that PFKL protein expression in carcinoma tissues was higher than that in surrounding tissues among paraffin sections of 456 HCC patients we collected (Supplementary Fig. 1b). Kaplan–Meier analysis indicated that overexpression of PFKL was correlated with poor overall survival of HCC patients from Sun Yat-Sen Memorial Hospital (log-rank = 4.234, *P* = 0.039) (Supplementary Fig. 1c) and Sun Yat-Sen University Cancer Center (log-rank = 4.437, *P* = 0.0352) (Supplementary Fig. 1d).

In summary, our study demonstrated that YTHDF3 promotes glycolysis by enhancing phosphofructokinase PFKL expression at both mRNA and protein level.

### YTHDF3 suppressed the degradation of PFKL mRNA via m^6^A modification

In our previous results, we have found that YTHDF3 promotes PFKL expression at mRNA and protein levels. Since YTHDF3 is one of m^6^A binding proteins, we speculated whether YTHDF3 affected PFKL expression by m^6^A modification of PFKL mRNA.

RNA binding protein immunoprecipitation assays (RIP) showed there was a direct correlation between YTHDF3 and PFKL in mRNA level in Sk-Hep-1 and HepG2 cells (Fig. 7d). Next, we predicted the sites where PFKL mRNA might be m^6^A modified by YTHDF3 by using SRAMP (http://www.cuilab.cn/sramp) (Fig. 7c). MeRIP assays (Fig. 7e) were performed by using both wild-type (WT) and two mutant (Mut) PFKL plasmids at m^6^A modification site to further confirm the effect of YTHDF3 m^6^A modification on PFKL mRNA. In mutant PFKL plasmids, adenosine bases (A) in predicted m^6^A binding sites were replaced by guanine base (G) or thymine base (T) to eliminate the effect of m^6^A methylation, while WT plasmid contained intact m^6^A binding site (Fig. 7f). Expectedly, YTHDF3 enriched PFKL mRNA in PFKL mutant groups were decreased significantly (Fig. 7g), demonstrating that YTHDF3-guided m^6^A modification of PFKL mRNA is key for regulation of PFKL mRNA in Huh7 and Sk-Hep-1 cells. Furthermore, in the presence of actinomycin D (an agent that blocks synthesis of RNA), we found that YTHDF3 knockdown promoted the degradation of PFKL mRNA, whereas YTHDF3 overexpression suppressed the degradation of PFKL mRNA (Fig. 7h). In brief, we found that YTHDF3 inhibits PFKL mRNA degradation via m^6^A modification.

### PFKL positively promotes YTHDF3 expression at protein level

**Fig. 8:**
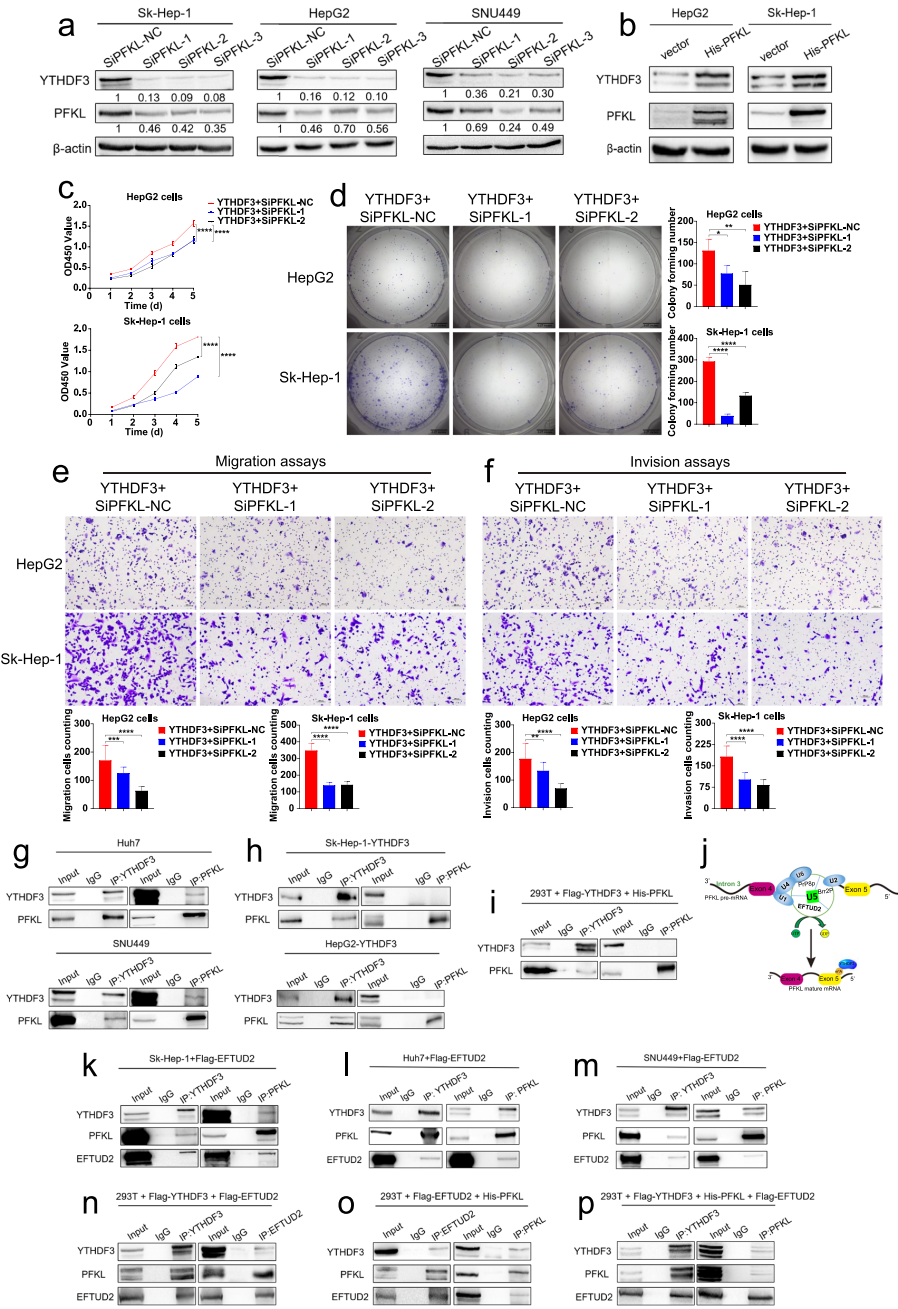
PFKL positively promotes YTHDF3 expression at protein level and PFKL interacts with YTHDF3 via EFTUD2 at the protein level. **a** PFKL and YTHDF3 protein expression by transiently transfecting small interfering RNA targeting PFKL mRNA into Sk-Hep-1, HepG2 and SNU449 cells for 12 hours. **b**. PFKL and YTHDF3 protein expression by transiently transfecting PFKL plasmids into Sk-Hep-1 and HepG2 cells for 12 hours. **c-f**. CCK8 proliferation assays, colony formation assays, transwell migration assays and transwell invasion assays after transiently transfecting small interfering RNA targeting PFKL into overexpression-YTHDF3 Sk-Hep-1, HepG2, SNU449 cells for 12 hours. The Image J software was used to quantified cells’ migration and invasion ability. Scale bar 200 μm. **g**. Endogenous co-immunoprecipitation assays were performed to investigate the interaction between YTHDF3 and PFKL with Huh7 and SNU449 cells. **h** Exogenous co-immunoprecipitation assays were performed to investigate the interaction between YTHDF3 and PFKL with YTHDF3-stably-overexpressed Sk-Hep-1 and HepG2 cells. **i** Co-immunoprecipitation assays were performed to investigate the interaction between YTHDF3 and PFKL after co-transfecting YTHDF3 and PFKL plasmids (10 μg respectively) into 293 T cells for 48 h. **j** The processes of PFKL pre-mRNA splicing and YTHDF3 m^6^A modification to PFKL mRNA. **k-m** Co-immunoprecipitation assays were performed to investigate the interaction between YTHDF3 and PFKL after transfecting EFTUD2 plasmids (10 μg) into Sk-Hep-1, Huh7 and SNU449 cells for 48 h. **n** Co-immunoprecipitation assays were performed to investigate the interaction between YTHDF3 and EFTUD2 after co-transfecting YTHDF3 and EFTUD2 plasmids (10 μg respectively) into 293 T cells for 48 h. **o** Co-immunoprecipitation assays were performed to investigate the interaction between PFKL and EFTUD2 after co-transfecting PFKL and EFTUD2 plasmids (10 μg respectively) into 293 T cells for 48 h **p** Co-immunoprecipitation assays were performed to investigate the interaction between YTHDF3 and PFKL after co-transfecting YTHDF3, PFKL and EFTUD2 plasmids (10 μg respectively) into 293 T cells for 48 h. Data are presented as mean ± SD (*p < 0.05, **p < 0.01, ***p < 0.001 and ****p < 0.0001).

It is reported that metabolic enzymes could have noncanonical functions in cancer and other pathologies [9]. As a key rate-limited enzyme, PFKL catalyzes conversion of fructose 6-phosphate to fructose 1,6-bisphosphate in glycolysis. However, the direct role of PFKL in m^6^A modification is unclear. We investigated whether PFKL could influence YTHDF3 expression in HCC, not as a key enzyme in glycolysis. After knocking down PFKL expression by transiently transfecting small interfering RNA targeting PFKL mRNA into Sk-Hep-1, HepG2 and SNU449 cells for 12 hours, we found that protein expression of YTHDF3 was decreased surprisingly (Fig. 8a). In contrast, we overexpressed PFKL protein by transiently transfecting PFKL plasmid into Sk-Hep-1 and HepG2 cells for 12 hours and found that protein expression of YTHDF3 was increased (Fig. 8b), indicating that YTHDF3 and PFKL have a positive loop regulation in HCC. Functionally, CCK8 proliferation assays, colony formation assays and transwell assays showed that PFKL knockdown successfully reversed the effects of YTHDF3 overexpression on proliferation (Fig. 8c, d), migration (Fig. 8e) and invasion ability (Fig. 8f) of Sk-Hep-1 and HepG2 cells. In short, PFKL positively regulates YTHDF3 expression at protein level.

### PFKL protein positively regulates YTHDF3 protein expression via inhibiting ubiquitination of YTHDF3 protein by EFTUD2

We found that YTHDF3 promotes PFKL mRNA expression via m^6^A modification as predicted and PFKL promotes YTHDF3 expression at protein level unexpectedly. To uncover the relationship between YTHDF3 and PFKL at protein level, we performed endogenous co-immunoprecipitation (Co-IP) assays with Huh7 and SNU449 cells and it seems that there was an interaction between YTHDF3 and PFKL at protein level (Fig. 8g). Unfortunately, there was no direct interaction between YTHDF3 and PFKL in exogenous Co-IP assays with YTHDF3-stably-overexpressed cells Sk-Hep-1 and HepG2 (Fig. 8h). Moreover, the Co-IP assay of YTHDF3 and PFKL plasmids co-transfection also confirmed that there was no direct interaction between these two proteins (Fig. 8i). These results suggested that PFKL might bind to YTHDF3 by the helps of other proteins.

It is well known that m^6^A is one of the most common RNA modifications and crucial for maintaining RNA splicing, translation, stability, degradation and export [10]. Simultaneously, pre-mRNA splicing plays a vital important role in gene expression. Pre-mRNA splicing is divided into two parts including removing intron regions and joining coding exon sequences to form a mature mRNA for translation. The process is catalyzed by the spliceosome, which is constituted by U1, U2, U4/U6 and U5 small nuclear ribonucleoproteins (snRNPs). EFTUD2 is a core subunit of the U5 snRNP component and it is related to the translation elongation factor-2 [11]. Hence, we guessed that PFKL might interact with YTHDF3 via EFTUD2 at protein level (Fig. 8j).

**Fig. 9:**
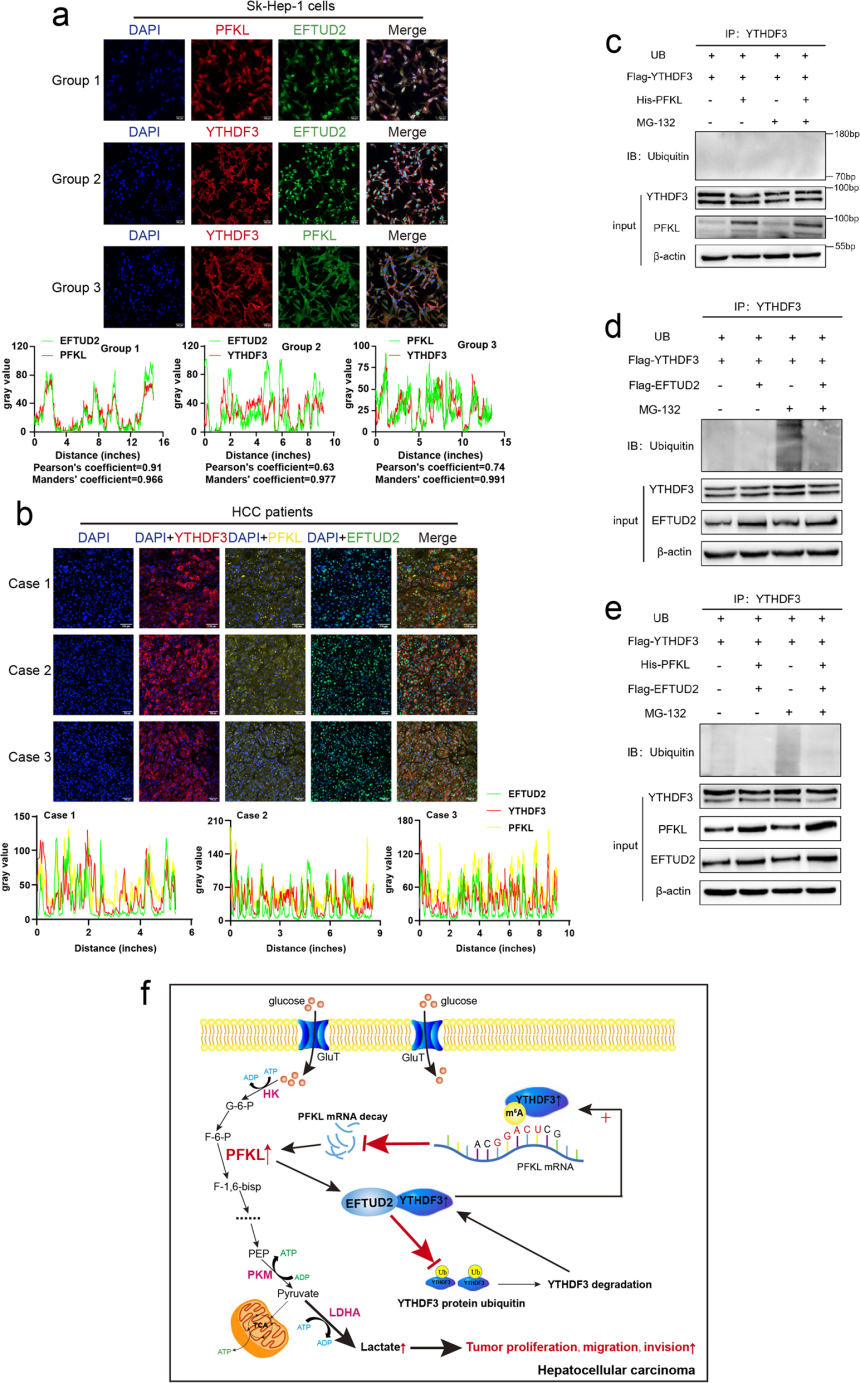
PFKL protein positively regulates YTHDF3 protein expression via inhibiting ubiquitination of YTHDF3 protein by EFTUD2. **a** Confocal assays of three group with Sk-Hep-1 cells incubated PFKL and EFTUd2 antibodies, YTHDF3 and EFTUd2 antibodies, or YTHDF3 and PFKL antibodies to investigate the localization between YTHDF3, PFKL and EFTUD2 (upper). Image J software was used to qualified the green, red and yellow fluorescence colocalization signal intensity, and calculated relative Pearson’s and Mander’s coefficients (lower). Scale bar 100 μm. **b** Multi-label immunofluorescence staining of different HCC patients to investigate the localization between YTHDF3, PFKL and EFTUD2 (upper). Image J software was used to qualified the green, red and yellow fluorescence colocalization signal intensity (lower). Scale bar 100 μm. **c** Ubiquitination assays of SNU449 cells co-transfected ubiquitin, YTHDF3 and PFKL plasmids. 20 nM MG-132 was added to RPMI1640 culture medium and cells were incubated continuously for 8 hours. **d**. Ubiquitination assays of SNU449 cells co-transfected ubiquitin, YTHDF3 and EFTUD2 plasmids. 20 nM MG-132 was added to RPMI1640 culture medium and cells were incubated continuously for 8 hours. **e** Ubiquitination assays of SNU449 cells co-transfected ubiquitin, YTHDF3, PFKL and EFTUD2 plasmids. 20 nM MG-132 was added to RPMI1640 culture medium and cells were incubated continuously for 8 hours. **f** Molecular mechanism of the relationship between YTHDF3, PFKL and EFTUD2 in HCC progression

Co-IP experiments demonstrated that there was an interaction between YTHDF3 and PFKL after EFTUD2 plasmid was transiently transfected into Sk-Hep-1, Huh7 or SNU449 cells (Fig. 8k, l, m). Then, YTHDF3 and EFTUD2 plasmids, or PFKL and EFTUD2 plasmids were co-transfected into 293 T cells respectively. We found that there was a direct interaction between YTHDF3 and EFTUD2 (Fig. 8n) and there was a direct interaction between PFKL and EFTUD2 (Fig. 8o). When YTHDF3, EFTUD2 and PFKL three plasmids were co-transfected into 293 T cells, PFKL and YTHDF3 were also immunoprecipitated together (Fig. 8p). What’s more, confocal assays of three group with Sk-Hep-1 cells incubated PFKL and EFTUD2 antibodies (Pearson’s coefficient = 0.91, Mander’s coefficient = 0.966), YTHDF3 and EFTUD2 antibodies (Pearson’s coefficient = 0.63, Mander’s coefficient = 0.977), or YTHDF3 and PFKL antibodies (Pearson’s coefficient = 0.74, Mander’s coefficient = 0.991) also indicated that there was co-location between YTHDF3, PFKL and EFTUD2 (Fig. 9a), and multi-label immunofluorescence staining of different HCC patients further demonstrated that PFKL interacted with YTHDF3 via EFTUD2 at the protein level (Fig. 9b). These results suggested that PFKL could bind to YTHDF3 by the help of EFTUD2. However, it has not reported whether EFTUD2 has a crucial role in the protein fate of YTHDF3.

Ubiquitination assays of SNU449 cells co-transfected ubiquitin, YTHDF3 and PFKL plasmids, or ubiquitin, YTHDF3 and EFTUD2 plasmids, or ubiquitin, YTHDF3, PFKL and EFTUD2 plasmids for 24 hours were performed to study the effect of EFTUD2 on ubiquitination of YTHDF3 protein. 20 nM MG-132 was added to RPMI1640 culture medium and cells were incubated continuously for 8 hours. We found that co-transfection of YTHDF3 and PFKL plasmids had no effect on ubiquitination of YTHDF3 protein, while the process of ubiquitination of YTHDF3 protein was decreased in the presence of EFTUD2 plasmid (Fig. 9c, d and e). Thus, we could conclude that PFKL protein positively regulated YTHDF3 protein expression via inhibiting ubiquitination of YTHDF3 protein by EFTUD2. These results indicated that EFTUD2 may act as a scaffold in protein-protein interaction and regulate as ubiquitin modification enzyme in YTHDF3 protein degradation which need to be investigated in the future.

## Discussion

Increasing evidences show that m^6^A modification of mRNA is critical for hepatocarcinogenesis and progression of HCC. Among them, a great mount of m^6^A “readers” such as YTHDC1/2, HNRNPA2B1, HNRNPC, HNRNPG, NKAP, IGF2BP1/2/3 and YTHDF1/2 are found to play significant roles in hepatocarcinogenesis and progression of HCC [12]. Nevertheless, the role of YTHDF3, a key reader in m^6^A modification in HCC is still unclear.

First of all, we found that YTHDF3 facilitates hepatocarcinogenesis and progression of HCC in vitro and in vivo. Clinically, YTHDF3 was overexpressed in carcinoma tissues among HCC patients and higher expression of YTHDF3 was correlated with poor prognosis of HCC patients. Functionally, YTHDF3 promoted proliferation, migration and invasion ability of HCC cells in vitro, and promoted xenograft tumor growth and lung metastasis in vivo. In addition, YTHDF3 contributed to hepatocarcinogenesis of mice induced by chemical carcinogens. At the same time, an analysis of the expression pattern of m^6^A regulator from Cancer Genome Atlas and Gene Expression Omnibus Database also shows that the expression of YTHDF3 is significantly increased in HCC tissues compared with normal tissues, and the high expression of YTHDF3 is closely related to the poor prognosis of HCC patients [13]. Moreover, highly-expressed lysine-specific demethylase 5B can promote proliferation, migration, invasion and other malignant phenotypes of HCC cells through miR-448 /YTHDF3/ITGA6 (Integrin subunit alpha 6) axis in hepatocellular carcinoma, confirming that YTHDF3 plays an important role in the occurrence and development of HCC [14]. These results are consistent with our findings. Interestingly, YTHDF3 was also found to play the key role in colorectal cancer [15] and breast cancer brain metastasis [16].

Next, we compared the global metabolic profiles of HCC tissues from *Ythdf3*^*+/+*^ and *Ythdf3*^*−/−*^ mice with untargeted metabolomics. We found that YTHDF3 knockout results in disorders of energy metabolic pathways, including glycolysis pathway in HCC. We found that the blood glucose level of high-expression YTHDF3 HCC patients was much higher than that in low-expression YTHDF3 HCC patients. In addition, the levels of blood glucose, pyruvate and lactate of liver tumor tissue of *Ythdf3*^*−/−*^ mice were obviously lower than those of *Ythdf3*^*+/+*^ mice in DEN and CCl_4_-induced HCC mice model. What’s more, at cellular level, YTHDF3 overexpression promoted glucose uptake, pyruvate and lactate production in HCC cells. As we know, metabolism reprogramming is a fundamental characteristic of cancers. The Warburg effect or aerobic glycolysis is a key reprogrammed metabolic pathway in cancers [17]. With respect to m^6^A modification and cancer aerobic glycolysis, it is reported that ALKBH5 enhances chemosensitivity of cells to cisplatin via glycolysis in bladder cancer [18], METTL3 overexpression enhances glycolysis metabolism and promotes tumor growth in HCC [19] and demethylase FTO can regulate PKM2 mRNA and promote its translation through m^6^A modification, thus promoting hepatocellular carcinoma tumorigenesis [20]. And our study suggests the key role of YTHDF3 in glucose metabolism of HCC.

Moreover, we found that YTHDF3 as a m^6^A “reader” protein, inhibits a key rate-limited enzyme of glycolysis PFKL mRNA degradation via m^6^A modification. Our MeRIP-qPCR assays demonstrated that YTHDF3 regulated PFKL expression at mRNA level via m^6^A modification, and RNA stability assays showed that YTHDF3 overexpression suppressed the degradation of PFKL mRNA. Nowadays, it is generally believed that YTHDF3 can facilitate translation of its target RNA interacting with YTHDF1, and affect methylated mRNA decay mediated through YTHDF2 [21]. It is reported that in hepatocellular carcinoma, circ-KIAA1429 promoted the progression of HCC by inhibiting the degradation of Zeb1 mRNA through the m^6^A-YTHDF3-Zeb1 axis [22], and in our study, YTHDF3 promotes the expression of PFKL by inhibiting degradation of PFKL mRNA, thereby promoting progression of HCC. Hence, our study establishes the functional connection between m^6^A “reader” protein YTHDF3 and rate-limited enzyme of glycolysis PFKL in HCC. What’s more, we also found that the expression of PFKL is significantly increased in carcinoma tissues in HCC and high expression of PFKL is associated with poor prognosis of HCC patients, supporting the key role of PFKL in HCC suggested by other studies [23, 24].

More importantly, we found that PFKL, a key rate-limited enzyme of glycolysis regulates m^6^A RNA modification by positively regulates YTHDF3 protein expression. Our results showed that protein expression of YTHDF3 was decreased after PFKL knockdown, and rescue experiments confirmed that PFKL knockdown reversed the effects of YTHDF3 overexpression on proliferation, migration and invasion ability of HCC cells. It has been widely believed that PFKL is a kind of kinase, physiologically catalyzing fructose 6-phosphate to fructose 1,6-bisphosphate in glycolysis. Interestingly, our study establishes a connection that PFKL reversely affects m^6^A modification “reader” YTHDF3 at protein level, not as a key rate-limited enzyme in HCC for the first time. In some cases, noncanonical functions of metabolic enzymes are closely associated with their canonical functions, while the non-canonical functions of metabolic enzymes often independently regulate processes that are highly relevant for cell transformation and cancer development, including promoting uncontrolled cell proliferation, inducing resistance to apoptosis or enhancing cell migration [25]. In recent years, more and more studies have shown that the rate-limited enzymes of glycolysis can regulate several non-glycolysis processes in the occurrence and progression of different cancers. For example, HK2 contributes to cell proliferation, migration, invasion, angiogenesis and drug resistance in HCC, and it can interact with voltage-dependent anion-selective channel protein 1 to inhibit cell apoptosis [26]. In addition, PKM2 can interact with transcription factors, such as HIF-1α, β-catenin/c-Myc, NF-κB and STAT3 [27, 28] to promote target genes transcription and enhance cancer cells growth and angiogenesis [29].

It is reported that m^6^A modification is involved in several processes of mRNA maturation, including splicing, nucleus export, translation, and degradation, like METTL3 regulates processing and metabolism of cellular RNAs by catalyzing the addition of m^6^A to mRNAs [30], and YTHDC1 recruits pre-mRNA splicing factor SRSF3 to bind regions of targeted mRNAs, directly regulating mRNA splicing [31]. Referring to EFTUD2, researchers have found that it can promote progression of HCC and its high expression indicates patients’ poor prognosis [32]. Herein, we found that PFKL protein positively regulates YTHDF3 protein expression via inhibiting ubiquitination of YTHDF3 protein by EFTUD2, forming a functional loop between pre-mRNA splicing process and m^6^A modification of mRNA. Through a series of endogenous and exogenous COIP assays, we confirmed that there were interactions between EFTUD2 with PFKL or YTHDF3 at protein level. Immunofluorescence assays also verified the co-localization of YTHDF3, PFKL and EFTUD2 at clinical and cellular levels. Ubiquitination assays demonstrated that co-transfection of YTHDF3 and PFKL plasmids had no effect on ubiquitination of YTHDF3 protein, while the process of ubiquitination of YTHDF3 protein was decreased in the presence of EFTUD2 plasmid, concluding that PFKL protein positively regulated YTHDF3 protein expression via inhibiting ubiquitination of YTHDF3 protein by EFTUD2. These results indicated that EFTUD2 may act as a scaffold in protein-protein interaction and regulate as ubiquitin modification enzyme in YTHDF3 protein degradation which need to be investigated in the future. Collectively, our study links pre-mRNA splicing process, m^6^A modification of mRNA and glycolysis in HCC, which may offer a promising approach for understanding the mechanism of HCC progression.

## Conclusion

In a word, our study found that YTHDF3 facilitates hepatocarcinogenesis and progression of HCC, and YTHDF3 overexpression predicts poor overall survival of HCC patients. In terms of mechanism, on the one hand, YTHDF3 is important for glycolysis metabolism of HCC, and it promotes the expression of PFKL at mRNA and protein levels. As a m^6^A reader, YTHDF3 inhibits PFKL mRNA degradation via m^6^A modification, accelerating aerobic glycolysis and progression of HCC. On the other hand, PFKL positively regulates the expression YTHDF3 at protein level, not as a kind of kinase, PFKL and YTHDF3 interact with each other by EFTUD2, and PFKL protein positively regulates YTHDF3 protein expression via inhibiting ubiquitination of YTHDF3 protein by EFTUD2, establishing a connection between pre-mRNA splicing and m^6^A modification in HCC (Fig. 9f).

## Supplementary Information

**Additional file 1.**

**Additional file 2.**

**Additional file 3: Supplementary Fig. 1.** a. Representative agarose gel electrophoresis image for distinguishing genotype of *Ythdf3*^*−/−*^, *Ythdf3*^*+/−*^, *Ythdf3*^*+/+*^ mice. b. Representative immunohistochemical images were shown (upper) and PFKL protein expression between surrounding and carcinoma tissues of HCC patients (*n* = 456) (lower). Scale bar 100 μm. c. Overall survival analysis of HCC patients with low and high expression of PFKL in Sun Yat-Sen Memorial Hospital (259 cases). d. Overall survival analysis of HCC patients with low and high expression of PFKL in Sun Yat-Sen University Cancer Center (245 cases). Arrows indicate the location of positive signal of PFKL protein.

**Additional file 4: Supplementary Fig. 2**. Global metabolic profiles of HCC tissues from *Ythdf3*^*+/+*^ and *Ythdf3*^*−/−*^ mice. a. OPLS-DA score showing separation of HCC tissues from *Ythdf3*^*+/+*^ and *Ythdf3*^*−/−*^ mice group. b. Fold changes of differential metabolites. c. Pathway analysis revealed that *Ythdf3*^*−/−*^ mice group made the greatest impact on the listed pathways. d. Network analysis and visualization using Cytoscape string.

**Additional file 5: Supplementary Table 1.** Correlation between YTHDF3 expression and clinicopathological characteristics in HCC patients.

**Additional file 6: Supplementary Table 2.** Correlation between YTHDF3 expression and biochemical indicators in 466 HCC patients.

## Data Availability

The data used to support the findings of this study are included within the article. All experiments involved in this study were repeated at least 3 times. Original data can be provided for reviewing.

## Abbreviations

YTHDF3: YTH domain family protein 3
PFKL: Phosphofructokinase, liver type
m^6^A: N^6^-methyladenosine
HCC: Hepatocellular carcinoma
ATP: Adenosine triphosphate
MeRIP: Methylated RNA immunoprecipitation
COIP: Co-immunoprecipitation
EFTUD2: Elongation factor Tu GTP binding domain containing 2
SD: Standard deviation
IHC: Immunohistochemistry
qPCR: Real-time quantitative polymerase chain reaction
shRNA: Short hairpin RNA
AFP: α-fetoprotein
CRISPR/Cas9: Clustered regularly interspaced short palindromic repeats/CRISPR associated 9
DEN: Diethylnitrosamine
CCl_4_: Carbon tetrachloride
ECAR: Extracellular acidification rate
OCR: Oxygen consumption rate
HK: Hexokinase
G-6-P: Glucose 6-phosphate
GPI: Phosphohexose isomerase
F-6-G: Fructose 6-phosphate
PFK1: Phosphofructokinase-1
ADP: Adenosine diphosphate
F-1,6-bisp: Fructose 1,6-biophosphate
ALDO: Aldolase
G3P: Glyceraldehyde 3-phosphate
GAPDH: Glyceraldehyde 3-phosphatedehydrogenase
1,3-PGA: 1,3-biphosphoglycerate
PGK: Phosphoglycerate kinase
3-PGA: 3-phosphoglycerate
PGAM: Phosphoglycerate mutase
2-PGA: 2-phosphoglycerate
ENO: Enolase
PEP: Phosphoenolpyruvate
PKM: Pyruvate kinase
LDH: Lactic dehydrogenase
PFKM: Phosphofructokinase, muscle type
PFKP: Phosphofructokinase, plasma type
RIP: RNA binding protein immunoprecipitation
SiRNA: Small interfering RNA
snRNPs: Small nuclear ribonucleoproteins
