# Supplementary material for: A functional loop between YTH domain family protein YTHDF3 mediated m6A modification and phosphofructokinase PFKL in glycolysis of hepatocellular carcinoma
Source: J Exp Clin Cancer Res. 2022 Dec 6;41:334. doi: 10.1186/s13046-022-02538-4 (PMC9724358; doi:10.1186/s13046-022-02538-4)
Supplement: Supplementary file 1 — Additional file 1. [file 13046_2022_2538_MOESM1_ESM.docx]

| Antibody | Company | Catalog Number | Experiments | Species | Concentration |
| --- | --- | --- | --- | --- | --- |
| YTHDF3 | Abcam | Ab103328 | Immunohistochemistry | Human, mice | 1:100 |
| YTHDF3 | Abcam | Ab220161 | Western blot | Human, mice | 1:1000 |
|  |  |  | IP | Human | 1:30 |
|  |  |  | [Immunofluorescence](javascript:;) | Human | 1:100 |
| PFKL | Immunoway | YT3685 | Immunohistochemistry | Human, mice | 1:200 |
|  |  |  | [Immunofluorescence](javascript:;) | Human | 1:50 |
| PFKL | Abcam | Ab241093 | Western blot | Human, mice | 1:2000 |
|  |  |  | IP | Human | 1:50 |
| EFTUD2 | Proteintech | 10208-1-AP | Western blot | Human | 1:1000 |
|  |  |  | [Immunofluorescence](javascript:;) | Human | 1:50 |
|  |  |  | IP | Human | 1:50 |
| EFTUD2 | Huabio | M1510-3 | [Immunofluorescence](javascript:;) | Human | 1:100 |
| HK2 | Proteintech | 66974-1 | Western blot | Human, mice | 1:10000 |
| PKM2 | Proteintech | 60268-1 | Western blot | Human, mice | 1:20000 |
| PFKM | Proteintech | 55028-1-AP | Western blot | Human, mice | 1:2000 |
| PFKP | Proteintech | 13389-1-AP | Western blot | Human, mice | 1:2000 |
| LDHA | Proteintech | 66287-1-Ig | Western blot | Human, mice | 1:2000 |
| Ki67 | CST | #12202 | Immunohistochemistry | Human, mice | 1:400 |
| IgG | Abclonal | AC005 | COIP | Human | 1:50 |
| Rb IgG/Alexa Fluor 488 | ZSGB-Bio | ZF-0511 | [Immunofluorescence](javascript:;) | Human | 1:300 |
| Goat IgG /Alexa Fluor 647 | ThermoFisher | A-21244 | [Immunofluorescence](javascript:;) | Human | 1:300 |

Supplementary 1---Antibody and Primers

1. Antibodies

2. qPCR Primers

| genes | Forward sequence | Reverse sequence |
| --- | --- | --- |
| Human YTHDF3 | TCAGAGTAACAGCTATCCACCA | GGTTGTCAGATATGGCATAGGCT |
| Human beta actin | GGACTTCGAGCAAGAGATGG | AGCACTGTGTTGGCGTACAG |
| Human PFKL | GGAGAAGCTGCGCGAGGTTTAC | ATTGTGCCAGCATCTTCAGCATGAG |
| Human EFTUD2 | AACAAGATTGACCGGCTGAT | CCAAAGGTGTCGGCATAGAT |
| Human HK2 | GCCATCCTGCAACACTTAGGGCTTGAG | GTGAGGATGTAGCTTGTAGAGGGTCCC |
| Human PKM2 | GCCCGTGAGGCAGAGGCTGC | TGGTGAGGACGATTATGGCCC |
| Human PFKM | GACTCCGAGCTGCCTACAAC | GCTGGACTTCGTAGCCTCCT |
| Human PFKP | CCAATTGCAACGTAGCTGTC | ACATCTGTCCAGCCGATTTC |
| Human LDHA | GGATCTCCAACATGGCAGCCTT | AGACGGCTTTCTCCCTCTTGCT |
| MeRIP-PFKL | GGACCTACTCGCACCTGAAC | GAAGGTCCTCTGGTGGCTCT |

| genes | Forward sequence | Reverse sequence |
| --- | --- | --- |
| mice YTHDF3 | GGTCAGTGGTAAAGGCTCCA | TGAGACTGCACTTGGTGAGG |
| mice beta actin | GGCTGTATTCCCCTCCATCG | CCAGTTGGTAACAATGCCATGT |
| mice PFKL | AGGGTCAGGTGCAAGAAGTG | GGCGTGGATGTTGTAGGTG |

3. Primers for genotypic identification of mice

| primer | 5’-3’ |
| --- | --- |
| F1-YTHDF3 | CTGGATTTGTACTCCCAGGACTGCC |
| R1-YTHDF3 | TAATACCCTTTCTGTCCAGCATTCA |
| R2-YTHDF3 | ATGGAAATACAACACCAGGCCCAAC |

4. Construction of YTHDF3 overexpression and knockdown cell lines.

|  | 5’-3’ |
| --- | --- |
| Lentivirus-YTHDF3 | YTHDF3 transcript 1 |
| Sh-NC | TTCTCCGAACGTGTCACGT |
| ShYTHDF3-1 | GCAATGATACTTTGAGTAAGG |
| ShYTHDF3-2 | GCAGTGGTATGACTAGCATTG |

5. SiRNA and plasmids

| SiRNA | Forward sequence | Reverse sequence |
| --- | --- | --- |
| Si-NC | UUCUCCGAACGUGUCACGUTT | ACGUGACACGUUCGGAGAATT |
| Si-YTHDF3-1 | AGAUGGUGUAUUUAGUCAACC | UUGACUAAAUACACCAUCUGG |
| Si-YTHDF3-2 | GGUGGAUUUCACCAGUUAAUG | UUAACUGGUGAAAUCCACCAA |
| Si-PFKL-1 | GCAUUUAUGUGGGUGCCAAUU | UUGGCACCCACAUAAAUGCUU |
| Si-PFKL-2 | GGUAAGAUCUCAGAGACUACA | UAGUCUCUGAGAUCUUACCUU |
| Si-PFKL-3 | GCAUCGUCAUGUGUGUCAUCC | AUGACACACAUGACGAUGCAG |

| Plasmid | Source |
| --- | --- |
| pCDH-CMV-MCS-EF1-Puro-YTHDF3 | Shanghai Integrated Biotech Solutions Co.,Ltd |
| PFKL pcDNA3.1-6xHIS-C | Shanghai Integrated Biotech Solutions Co.,Ltd |
| EFTUD2 pcDNA3.1-3xFlag-C | Shanghai Integrated Biotech Solutions Co.,Ltd |
