# Supplementary material for: A functional loop between YTH domain family protein YTHDF3 mediated m6A modification and phosphofructokinase PFKL in glycolysis of hepatocellular carcinoma
Source: J Exp Clin Cancer Res. 2022 Dec 6;41:334. doi: 10.1186/s13046-022-02538-4 (PMC9724358; doi:10.1186/s13046-022-02538-4)

The detail information about *Ythdf3^-/-^* C57BL/6 mouse.

1. Mouse Information

| Name | C57BL/6-Ythdf3^tm1cyagen^ |
| --- | --- |
| Serial Number | KOCMP-06061-Ythdf3 |
| Gene | Ythdf3 |
| NCBI ID | 229096 |
| Strain | C57BL/6 |
| Type | Conventional knockout |

2. gRNA target sequence

gRNA1 (matching forward strand of gene): AGTCACAAATAGTTACTTGAAGG

gRNA2 (matching forward strand of gene): AAACATATACTGTGAAGCGTTGG

3. Delivery Information


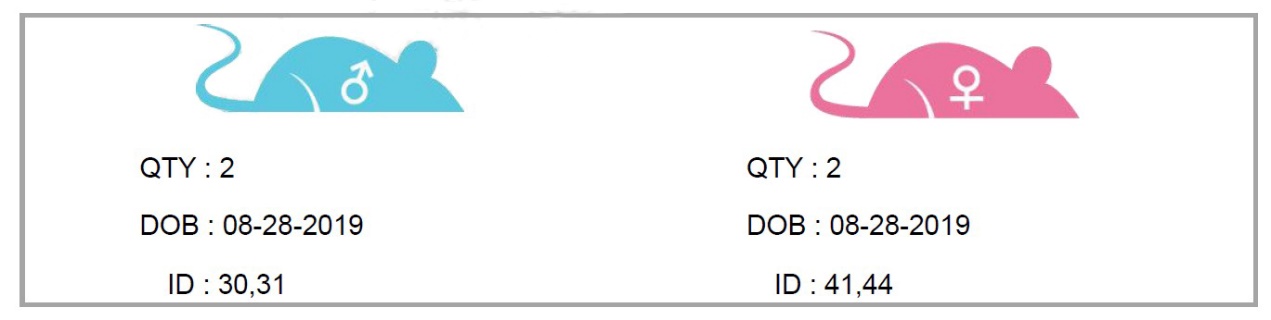


4. Genotyping Strategy


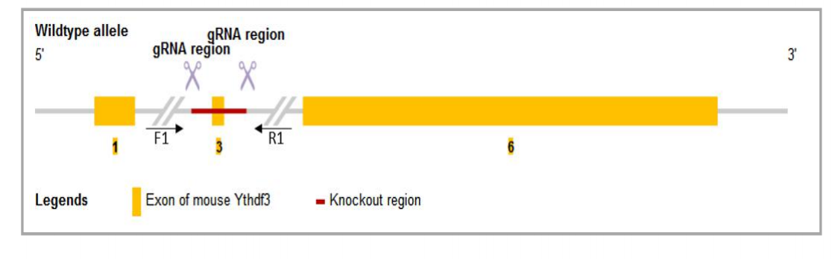

Supplement: Supplementary file 2 — Additional file 2. [file 13046_2022_2538_MOESM2_ESM.docx]
