# Supplementary figures and images for: A functional loop between YTH domain family protein YTHDF3 mediated m6A modification and phosphofructokinase PFKL in glycolysis of hepatocellular carcinoma
Source: J Exp Clin Cancer Res. 2022 Dec 6;41:334. doi: 10.1186/s13046-022-02538-4 (PMC9724358; doi:10.1186/s13046-022-02538-4)

**a**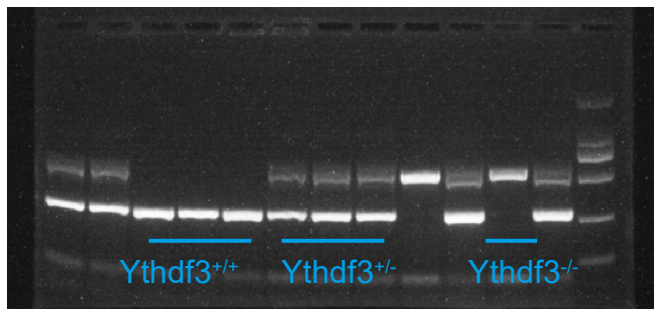**b**

Surrounding tissue

Carcinoma tissue

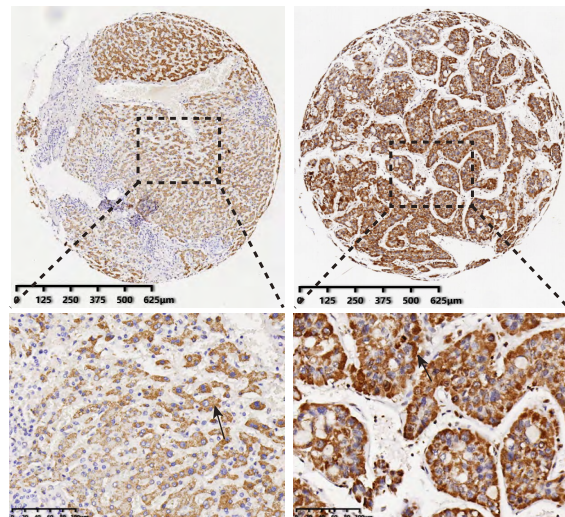**c**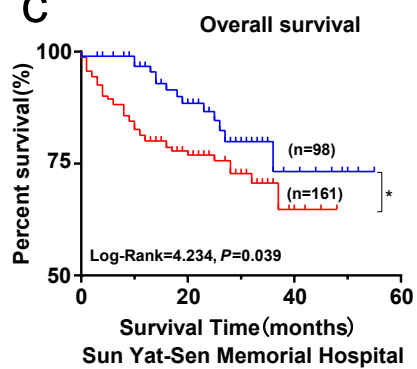**d**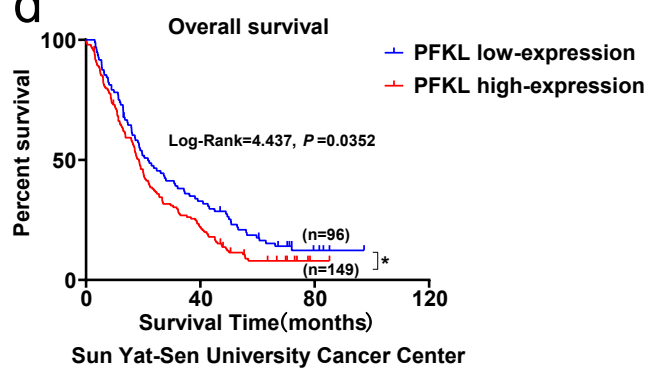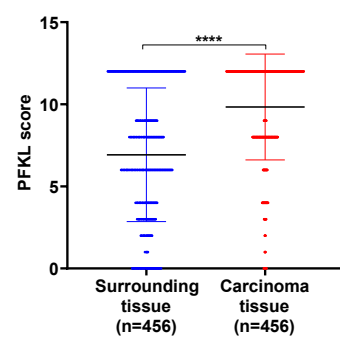

Supplement: Supplementary file 3 — Additional file 3: Supplementary Fig. 1. a. Representative agarose gel electrophoresis image for distinguishing genotype of Ythdf3−/−, Ythdf3+/−, Ythdf3+/+ mice. b. Representative immunohistochemical images were shown (upper) and PFKL protein expression between surrounding and carcinoma tissues of HCC patients (n = 456) (lower). Scale bar 100 μm. c. Overall survival analysis of HCC patients with low and high expression of PFKL in Sun Yat-Sen Memorial Hospital (259 cases). d. Overall survival analysis of HCC patients with low and high expression of PFKL in Sun Yat-Sen University Cancer Center (245 cases). Arrows indicate the location of positive signal of PFKL protein. [file 13046_2022_2538_MOESM3_ESM.pdf]

### Scores Plot

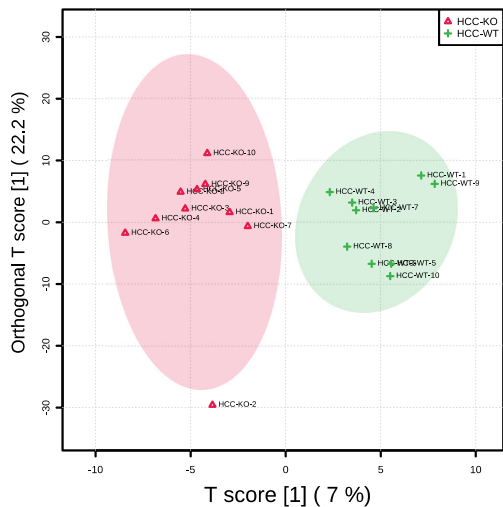

**b**

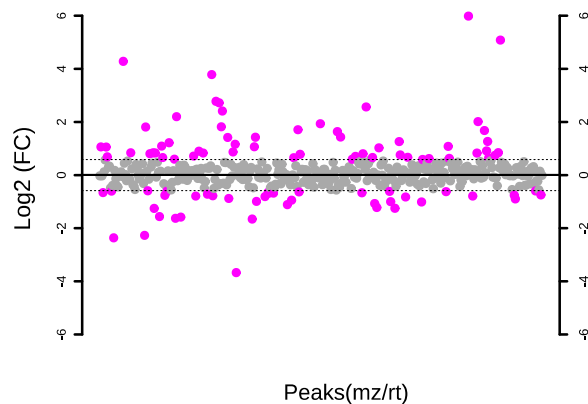

C

### Enrichment Overview (top 25)

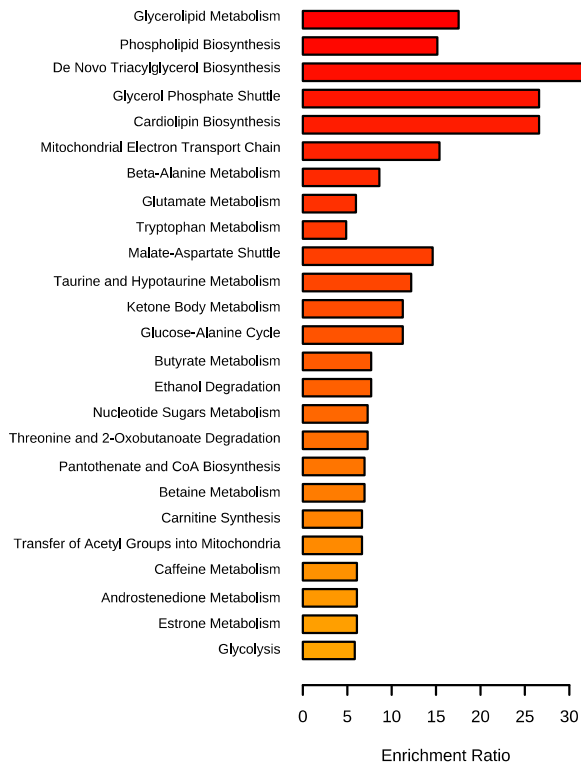

d

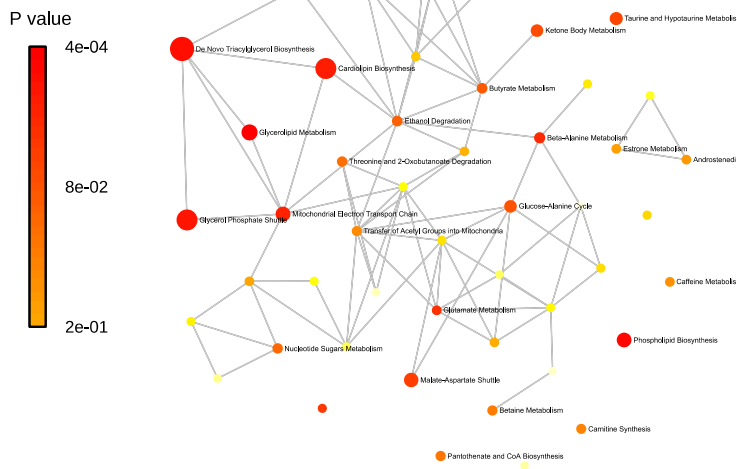

Supplement: Supplementary file 4 — Additional file 4: Supplementary Fig. 2. Global metabolic profiles of HCC tissues from Ythdf3+/+ and Ythdf3−/− mice. a. OPLS-DA score showing separation of HCC tissues from Ythdf3+/+ and Ythdf3−/− mice group. b. Fold changes of differential metabolites. c. Pathway analysis revealed that Ythdf3−/− mice group made the greatest impact on the listed pathways. d. Network analysis and visualization using Cytoscape string. [file 13046_2022_2538_MOESM4_ESM.pdf]
