# Supplementary material for: A functional loop between YTH domain family protein YTHDF3 mediated m6A modification and phosphofructokinase PFKL in glycolysis of hepatocellular carcinoma
Source: J Exp Clin Cancer Res. 2022 Dec 6;41:334. doi: 10.1186/s13046-022-02538-4 (PMC9724358; doi:10.1186/s13046-022-02538-4)
Supplement: Supplementary file 5 — Additional file 5: Supplementary Table 1. Correlation between YTHDF3 expression and clinicopathological characteristics in HCC patients. [file 13046_2022_2538_MOESM5_ESM.docx]

| Features | | YTHDF3 low expression | YTHDF3 high expression | *P* value |
| --- | --- | --- | --- | --- |
| Age (years) | | 54.92±10.89 | 54.70±11.86 | 0.840 |
| Gender | male | 151 | 257 | 0.099 |
|  | female | 28 | 30 |  |
| Drinking/Smoking history | without | 120 | 194 | 0.901 |
|  | with | 59 | 93 |  |
| Family history | without | 155 | 259 | 0.223 |
|  | with | 24 | 28 |  |
| HBV infection | without | 27 | 39 | 0.653 |
|  | with | 152 | 248 |  |
| Liver cirrhosis | without | 91 | 164 | 0.184 |
|  | with | 88 | 123 |  |
| Degree of differentiation | poor | 62 | 107 | 0.806 |
|  | moderate | 94 | 149 |  |
|  | well | 22 | 31 |  |
| Tumor largest diameter (cm) | | 6.06±3.94 | 6.98±4.61 | 0.023* |
| Tumor number | solitary | 125 | 189 | 0.354 |
|  | multiple | 53 | 97 |  |
| TNM stage | Ⅰ+Ⅱ | 135 | 191 | 0.038* |
|  | Ⅲ+Ⅳ | 43 | 95 |  |
| Capsular invasion | without | 68 | 82 | 0.034* |
|  | with | 111 | 205 |  |
| Vascular invasion | without | 102 | 132 | 0.021* |
|  | with | 77 | 155 |  |
| Lymph node metastasis | without | 172 | 275 | 0.886 |
|  | with | 7 | 12 |  |
| Distant metastasis | without | 111 | 177 | 0.481 |
|  | with | 40 | 75 |  |
| Relapse | without | 78 | 150 | 0.118 |
|  | with | 72 | 100 |  |

Supplementary Table 1 Correlation between YTHDF3 expression and clinicopathological characteristics in HCC patients

1. Quantitative data are mean ± SD.

2. The *t* test was used among the quantitative data. Chi-square tests were used among the qualitative data.

3. **P* < 0 .05
