# Supplementary material for: A functional loop between YTH domain family protein YTHDF3 mediated m6A modification and phosphofructokinase PFKL in glycolysis of hepatocellular carcinoma
Source: J Exp Clin Cancer Res. 2022 Dec 6;41:334. doi: 10.1186/s13046-022-02538-4 (PMC9724358; doi:10.1186/s13046-022-02538-4)
Supplement: Supplementary file 6 — Additional file 6: Supplementary Table 2. Correlation between YTHDF3 expression and biochemical indicators in 466 HCC patients. [file 13046_2022_2538_MOESM6_ESM.docx]

Supplementary Table 2 Correlation between YTHDF3 expression and biochemical indicators in 466 HCC patients

|  | YTHDF3 low expression | YTHDF3 high expression | *P* value |
| --- | --- | --- | --- |
| Blood glucose (mmol/L) | 5.31±1.05 | 5.95±2.44 | 0.0001*** |
| Blood LDHA (U/L) | 227.65±102.75 | 234.72±106.83 | 0.571 |
| AFP (ng/ml) | 5112.49±18455.97 | 16109.61±34547.08 | <0.0001*** |
| AST(U/L) | 59.37±67.75 | 60.77±56.50 | 0.809 |
| ALT(U/L) | 53.60±52.10 | 50.73±50.13 | 0.555 |
| TBIL(μmol/L) | 25.27±47.85 | 24.64±53.74 | 0.899 |
| DBIL(μmol/L) | 9.08±28.38 | 8.44±31.27 | 0.831 |
| IBIL(μmol/L) | 17.14±22.53 | 17.00±27.43 | 0.958 |
| ALP(U/L) | 123.36±80.62 | 123.37±98.18 | 0.999 |
| Albumin(g/L) | 39.58±5.17 | 39.82±5.05 | 0.634 |
| PT(s) | 12.93±1.13 | 12.85±1.09 | 0.475 |
| Fibrinogen(g) | 3.90±10.06 | 3.13±1.08 | 0.200 |
| APTT(s) | 28.09±4.20 | 28.23±4.48 | 0.732 |
| D-dimer | 1.43±2.63 | 1.09±1.53 | 0.121 |

1. Abbreviations: AFP alpha-fetal protein, AST aspartate transaminase, ALT alanine transaminase, TBIL total bilirubin,

DBIL direct bilirubin, IBIL indirect bilirubin, ALP alkaline phosphatase, PT prothrombin time,

APTT partial activated thromboplastin time.

2. All data are mean ± SD.

3. The *t* test or the rank sum test is chosen according to normality of the groups’ data

4. ****P*<0 .001
